# Evaluation of the prehospital use of a Valsalva assist device in the emergency treatment of supraventricular tachycardia (EVADE SVT): study protocol for a stepped wedge cluster randomised controlled trial

**DOI:** 10.1136/bmjopen-2023-073315

**Published:** 2023-06-08

**Authors:** Andrew Appelboam, Ria Osborne, Obioha Ukoumunne, Sarah Black, Suzanne Boot, Nicholas Richards, Natalie Scotney, Shelley Rhodes, Tim Cranston, Ruth Hawker, Annette Gillett, Ben Jones, Annie Hawton, Mark Dayer, Siobhan Creanor, EVADE SVT Study Collaborators, Michela Cox

**Affiliations:** 1 Medical School, University of Exeter, Exeter, UK; 2 Academic Department of Emergency Medicine, Royal Devon University Healthcare NHS Foundation Trust, Exeter, Devon, UK; 3 Research, Audit and Improvement, South Western Ambulance Service NHS Foundation Trust, Exeter, UK; 4 NIHR ARC South West Peninsula (PenARC), University of Exeter, Exeter, UK; 5 South Western Ambulance Service NHS Foundation Trust, Exeter, UK; 6 Exeter Clinical Trials Unit, University of Exeter Medical School, Exeter, UK; 7 PPI Representative, honorary SWASFT contract, South Western Ambulance Service NHS Foundation Trust, Exeter, UK; 8 Peninsula Childhood Disability Research Unit (PenCRU), University of Exeter Medical School, Exeter, UK; 9 Health Economics Group, University of Exeter, Exeter, Devon, UK; 10 Cardiology, Taunton and Somerset NHS Foundation Trust, Taunton, UK; 11 Faculty of Health, University of Plymouth, Plymouth, UK

**Keywords:** ACCIDENT & EMERGENCY MEDICINE, Pacing & electrophysiology, Adult cardiology

## Abstract

**Introduction:**

Patients with episodes of supraventricular tachycardia (SVT), a common heart arrhythmia, are often attended by ambulance services. International guidelines advocate treatment with the Valsalva manoeuvre (VM), but this simple physical treatment has a low success rate, with most patients requiring conveyance to hospital. The Valsalva Assist Device (VAD) is a simple device that might help practitioners and patients perform a more effective VM and reduce the need for patients to be taken to hospital.

**Methods and analysis:**

This stepped wedge cluster randomised controlled trial, conducted within a UK ambulance service, compares the current standard VM with a VAD-delivered VM in stable adult patients presenting to the ambulance service with SVT. The primary outcome is conveyance to hospital; secondary outcomes measures include cardioversion rates, duration of ambulance care and number of subsequent episodes of SVT requiring ambulance service care. We plan to recruit approximately 800 patients, to have 90% power to detect an absolute reduction in conveyance rate of 10% (from 90% to 80%) between the standard VM (control) and VAD-delivered VM (intervention). Such a reduction in conveyance would benefit patients, the ambulance service and receiving emergency departments. It is estimated potential savings would pay for devices for the entire ambulance trust within 7 months.

**Ethics and dissemination:**

The study has been approved by the Oxford Research Ethics Committee (reference 22/SC/0032). Dissemination will be through peer-reviewed journal publication, presentation at national and international conferences and by the Arrhythmia Alliance, a patient support charity.

**Trial registration number:**

ISRCTN16145266.

**Strengths and limitations of this study**

- The efficient, pragmatic design of the trial and use of routine data, enables delivery of the study during normal ambulance service care of patients with supraventricular tachycardia, as the device is brought into standard use.
- The study is designed to detect a difference in conveyance rate; a reliable outcome that is of importance to patients, the ambulance service and receiving hospitals.
- Although individual participant randomisation and blinding is not possible in this study, blinding of the trial statisticians will be maintained until the statistical analysis plan is finalised and signed off by the Trial Steering Committee.

## Introduction

Supraventricular tachycardias (SVT) are a group of common heart rhythm disorders causing episodes of fast heart rates. The incidence of SVT is estimated to be 35 episodes per 100 000 persons/year^1^ and attacks are unpleasant and disruptive to patients’ lives.^2^ SVT often occurs because of an additional electrical pathway in the heart, causing a ‘re-entrant tachycardia’.

Prehospital emergency services often attend patients with attacks of SVT. A regional Australian ambulance service recorded seeing 800 cases of SVT in a year^3^ with audit data suggesting a similar number of episodes in the South Western Ambulance Service NHS Foundation Trust (SWASFT), in the South West of England, UK. International ambulance treatment guidelines recommend initial treatment with the Valsalva manoeuvre (VM).^4 5^ This simple physical treatment involves an exhalation strain against resistance which causes a slowing of the heart (mediated by the vagal nerve) and can cardiovert an SVT back to normal sinus rhythm. Unfortunately, cardioversion rates in routine prehospital practice can be as low as 5% and most patients are conveyed to hospital with ongoing SVT.^6 7^

Results from our feasibility study suggested SWASFT ambulance clinicians treat around 500 of the SVT episodes they see each year, using a VM with approximately 90% of these patients being conveyed to hospital.^8^ This ambulance service has previously adopted the modified Valsalva manoeuvre (mVM) into its guidelines as the first-line treatment in stable patients with SVT. This guideline, in common with others, recommends that the VM strain is delivered by blowing into a 10 mL syringe^9^ followed by an internationally recommended^10^ postural modification which has shown to improve cardioversion rates.^11^ However, syringes are unreliable in generating the correct VM strain pressure^12^ and remembering the modified manoeuvre may be difficult for individual clinicians, who only occasionally see these patients.

**Figure 1:**
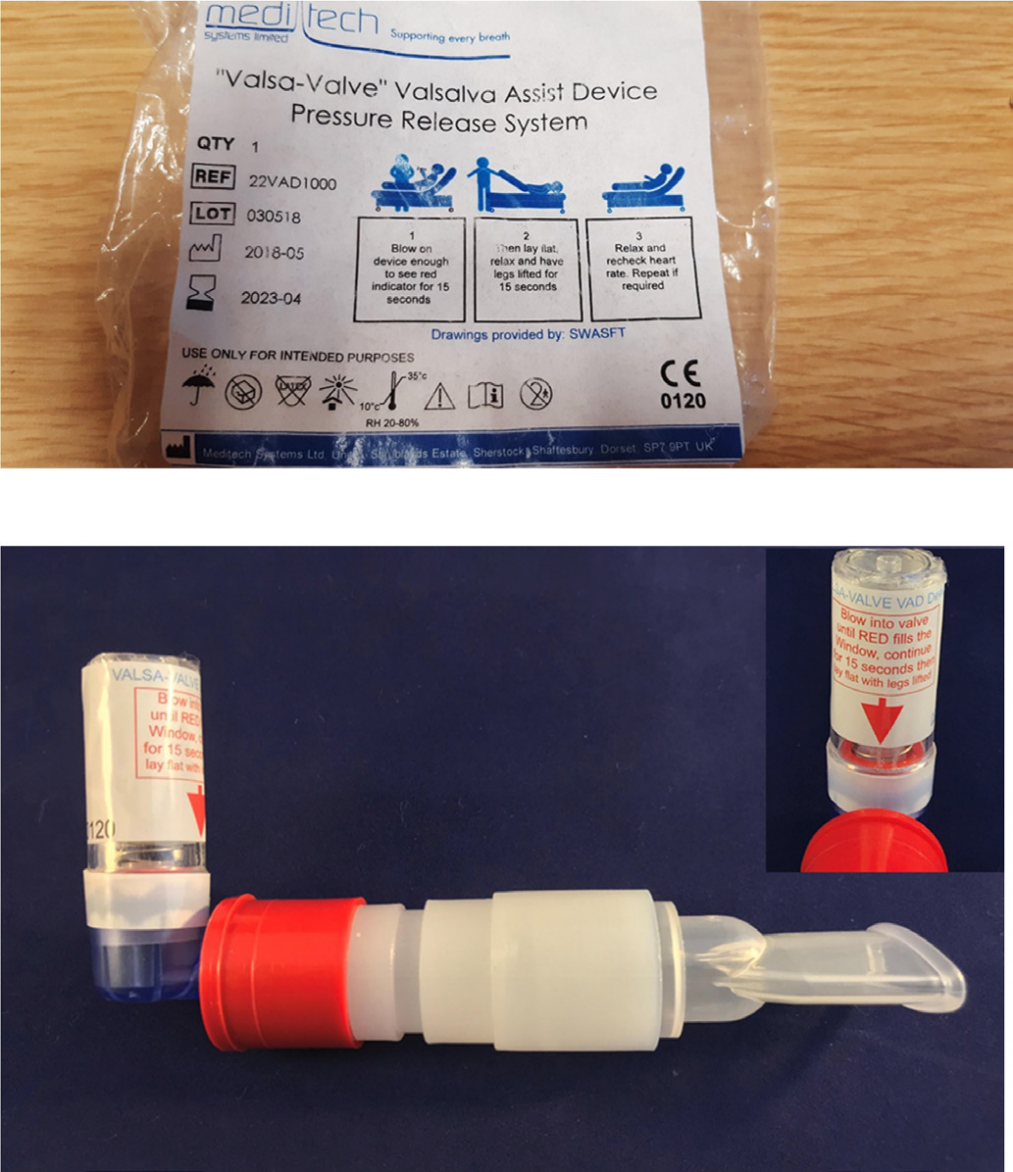
The ‘Valsa-Valve’ Valsalva Assist Device packaging and device details. CE, Conformité Européene; QTY, quantity; REF, reference number; RH, relative humidity; SWASFT, South Western Ambulance Service NHS Foundation Trust.

A simple, easy to use Valsalva Assist Device (VAD) has been developed which provides the correct, recommended resistance (40 mm Hg) for the VM exhalation strain.^13^ A Conformité Européene (CE) marked VAD has been produced by Meditech Systems Limited (www.meditechsystems.co.uk), marketed as the ‘Valsa-Valve’ VAD (see figure 1). This VAD includes instructions for use on the device and it’s packaging, including information on the timings and the postural changes required for the mVM. If appropriate, these single-patient-use devices can also be left with the patient, for subsequent use should an attack recur. The VAD might improve the quality of the VM and increase the chances of cardioversion and non-conveyance compared with the standard approach using syringe delivered VMs. Reducing ambulance conveyance of patients to hospital, when appropriate and possible, is increasingly important to health services and patients.^14^

A definitive randomised controlled trial is needed to test whether the availability of a VAD to ambulance clinicians, when treating patients with SVT, affects the success of treatment and the need for conveyance to hospital, compared with current practice. A recent feasibility study and patient and public feedback have informed the nature and design of the definitive trial outlined in this protocol paper.

## Methods and analysis

### General study design and conduct

This study is an efficient, pragmatic, stepped wedge cluster randomised controlled trial to see if the routine availability of a VAD and its instructions (intervention), to treat adult patients presenting to SWASFT ambulance clinicians with SVT, can reduce the rate of hospital conveyance compared with standard practice (control). The stepped wedge design involves the allocation of ambulance service clusters across the whole service. These clusters all start as controls, providing current standard care, before being randomly assigned to using the VAD, as a new standard treatment, in defined groups and periods over the duration of the study (further details provided below). This study protocol is reported according to Standard Protocol Items: Recommendations for Interventional Trials reporting guidelines.^15^

### Study population and setting

Eligible patients presenting to SWASFT with suspected SVT and who are treated with a VM by an SWASFT ambulance clinician, will be included. The study will be conducted entirely within SWASFT, UK. This ambulance service uses a searchable electronic patient care record (ePCR) as standard when attending patients. This was shown to be a reliable and easily searchable data source in our feasibility study. SWASFT also has an established clinical guideline advocating the use of an mVM and non-conveyance of successfully treated SVT patients.

The study aims to include all adult patients aged 18 years and over for whom there is a record of a VM delivered by an ambulance clinician, working out of a participating ambulance station, for presumed SVT.

Patients who subsequently withdraw consent for use of their data or have an existing general opt-out for use of routine National Health Service (NHS) data for research will be excluded from the trial. Data from patients who are prisoners at the time of treatment, patients with permanent lack of capacity as recorded by the treating clinician and patients solely treated by an SWASFT member of staff outside of the included ambulance station clusters (eg, air ambulance, Hazardous Area Response Team, British Association for Immediate Care doctor) will also be excluded. Data from patients previously included in the study will be excluded from the primary outcome analysis but will be used in analyses of some secondary outcomes. A summary table of the trial eligibility criteria is shown in table 1.

**Table 1:** Trial inclusion and exclusion criteria

Inclusion criteria	Adult patients (age 18 years or over). Record of a Valsalva manoeuvre for presumed SVT having been delivered by an ambulance clinician assigned to a participating ambulance station cluster.
Exclusion criteria	Subsequent withdrawal of patient consent to use their data. Existing opt-out for use of NHS data for research. Data from patients who are prisoners at the time of treatment. Patients with permanent lack of capacity as recorded by the treating clinician. Patients solely treated by an SWASFT resource not from one of the included ambulance station clusters, for example, air ambulance, Hazardous Area Response Team, Tiverton Minor Injuries Unit, British Association for Immediate Care doctor. Data from patients previously included in the study will be excluded from the primary outcome analysis but will be used in analyses of some secondary outcomes.

NHS, National Health Service; SVT, supraventricular tachycardia; SWASFT, South Western Ambulance Service NHS Foundation Trust.

### Study procedures

Potential study participants will undergo standard clinical assessment by ambulance clinicians. For suspected SVT this typically includes medical history, recording of routine initial observations of pulse, blood pressure, respiratory rate, oxygen saturation and the recording of a 12-lead ECG. Routinely collected clinical data are entered into the ePCR system and so are available for subsequent trial data extraction for eligible participants.

The screening of eligibility for VM treatment, the conduct of the VM and all other aspects of clinical care will be entirely governed by SWASFT clinical guidelines, interpreted and followed at the discretion of the treating clinician. On commencement of the intervention phase of the trial, SWASFT clinical guidelines will be updated to include the use of the VAD, where available as part of standard care.

### Trial treatment

The stocking of ambulances with the VAD (and thus the allocation of interventions) will be determined by the randomisation schedule as described below. This will ensure that a VAD is available for use by the treating clinician according to the randomisation schedule. The use of devices will thereafter be at the discretion of the treating clinician but encouraged through trial training and publicity; device use will be recorded and used as a measure of device acceptability.

Post-treatment assessment usually includes the recording of a further 12-lead ECG to determine whether normal sinus rhythm has been restored. However, this is not universal and often omitted if there has been no clear change in the heart rate and rhythm. SWASFT guidelines allow for non-conveyance in stable, well patients who have cardioverted, have normal observations and remain in sinus rhythm after 20 min of observation. In this case, patients are advised to seek further follow-up with their general practitioner. Patients will also be given the device (syringe or VAD) they used to perform the VM to take away, with written instructions, in case of recurrent SVT attacks.

### Consent

All patients will be given a participant information leaflet (PIL), specific to the stage of the trial, at a convenient time after treatment, at the discretion of the treating ambulance clinician. The front of the PIL will clearly state that the information can be read when the patient is ready and feels well enough. The PIL contains an explanation of SVT, the nature of the trial and how to opt out, should they not want their data to be used. Ambulance clinicians will be asked to record in the ePCR, confirmation that the PIL was given to the patient or a carer. As part of the screening and data entry process, the Lead Research Paramedic will confirm whether a PIL was given by checking the ePCR and, if not recorded, will contact the treating paramedic to confirm. If there is no evidence that a PIL was given at the time of treatment, the Lead Research Paramedic will post a PIL to the patient.

For patients with temporary lack of capacity (eg, due to their condition or associated illness) the PIL will be given to the next of kin or carer, if available, to explain and share with the patient when they are well enough. Patients with permanent severe lack of capacity, as determined by the treating ambulance clinician, will not be included. PIL translations will be sent to non-English speakers by SWASFT research staff where possible.

The PIL gives details of how patients can opt out of their routine data being used in the trial. This can be through immediate verbal opt-out (to the treating ambulance clinician) or retrospectively by telephone, post or email. Immediate verbal opt-outs will be recorded on the ePCR and screening of cases for inclusion in the trial data set will include a full search for opt-outs in the ePCR or received by any other means. An opt-out received at any time during the trial will render the patient ineligible, and their clinical data for their initial and subsequent episodes will not be included in the study. Safety follow-up data, already recorded up to the point of withdrawal, will be kept.

In the UK, patients also have the option to nationally opt out of having any of their routine digital NHS data used for research. The national rate of this type of opt-out currently stands at around 5%. After taking advice from the regional research ethics committee and our public and patient advisory group, we have elected to exclude all patients with a national data opt out unless the patient advises that they want to be included in the study (details of how to do this are included in the PIL).

In the absence of (i) the patient having a national opt-out and (ii) the research team receiving any individual opt-out, inferred consent for routine data use will be assumed. There is a precedent for this approach to collecting routine data for emergency medicine research and it is considered to constitute active recruitment as stated in the National Institute for Health and Care Research (NIHR) Clinical Research Network (CRN) recruitment policy document.^16^

Individual trial participation will last from the start of the SVT ambulance service care episode until 3 months after this time to enable the collection of further ambulance service use data as part of the planned recurrent SVT and safety follow-up period.

### Health-related quality of life data

The PIL also includes an invitation to an optional questionnaire to collect (non-routine) data on the effects of the participant’s condition and ambulance service treatment on their health-related quality of life. The invitation includes a web link (with a QR code) to this part of the study, printed on the PIL. Those who choose to participate will be given further information and asked for consent if they follow this link. Once they have given consent, they will be asked to complete the EQ-5D-5L (EuroQol health related quality of life instrument) questionnaire online, ideally within a week of the patient feeling well enough to complete the form; this will also be explained in the PIL. Participants who consent to this study component will be contacted digitally and reinvited to complete the EQ-5D-5L, 3 months after the SVT ambulance service care episode. Data from completed forms will be linked with the study database entry for the index presentation. Paper or emailed copies of the EQ-5D-5L will be sent to participants if requested, as outlined in the PIL. Paper or online EQ-5D-5L will be used under licence.

### Formation of ambulance station clusters and randomisation

Ambulance station clusters will be formed from the 93 SWASFT ambulance stations in existence at the time of study set-up. Recent historical data on activity and general conveyance rates for all stations were collected to inform the formation of trial clusters and sample size calculations. After merging very small and closely located stations, while maintaining the largest possible number of similar sized clusters, 80 (mostly single station) ambulance station clusters were identified. Clinicians working from these stations use equipment bags strictly belonging to and stocked by that station.

The stepped wedge trial design is comprised of five stages, with each stage lasting 4 months. In the first stage, none of the station clusters will have access to the VAD and patients will be treated as per standard practice. The clusters will then be randomly allocated, by computer-generated sequences (stratified by location and balanced on cluster size), to include VADs with their equipment bags, in one of the four remaining stages, so that 20 clusters and their clinicians receive access to the VAD for the first time at each stage. Randomisation will be performed by an independent statistician otherwise unconnected with the trial.

**Figure 2:**
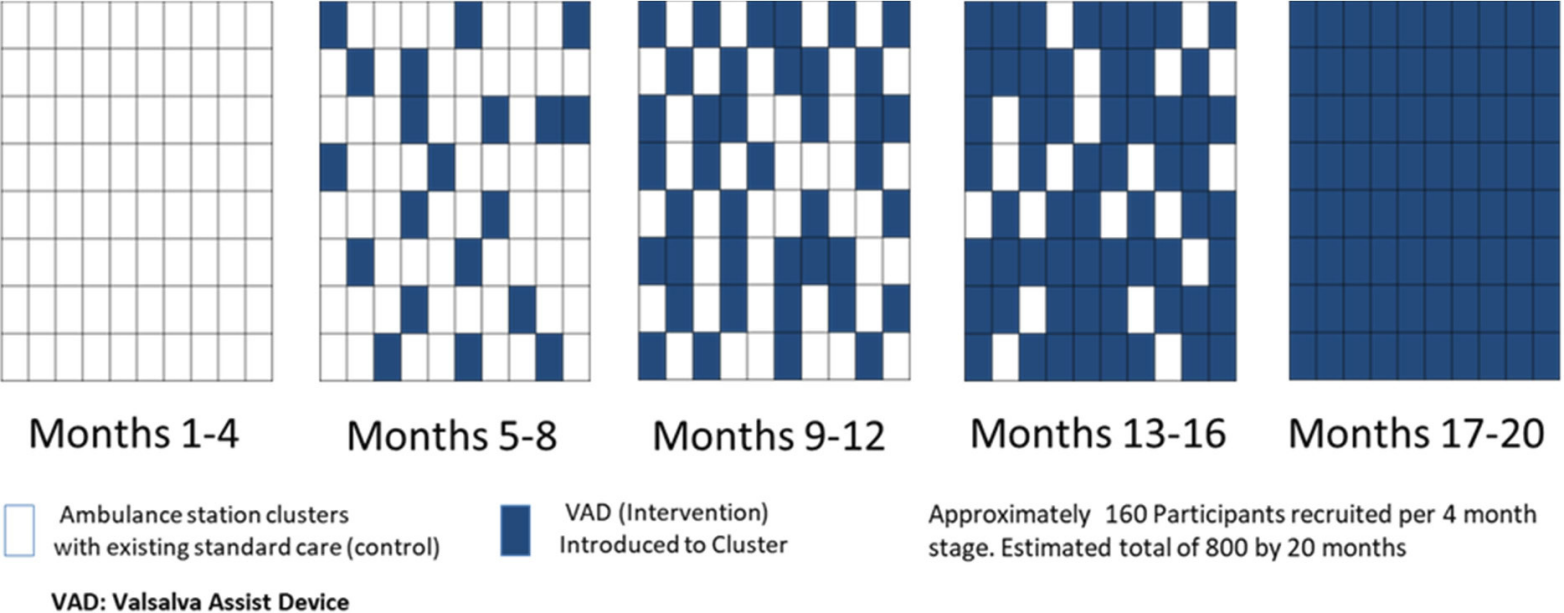
Scheme for the randomised stepped wedge introduction of the Valsalva Assist Device to the ambulance service.

Once the VAD has been introduced, the cluster will retain the use of devices for the remainder of the study so that by the fifth stage, all 80 clusters and their clinicians will have access to the VAD (see figure 2). Devices will be added to a clearly marked ‘Valsalva pouch’ attached to the standard equipment bag. These bags are checked and restocked daily and carried by all treating ambulances. All station clusters randomised to VAD use will initiate availability of the VAD on the same day, coordinated by SWASFT supply logistics and the local ambulance station ‘champions’ (listed as trial collaborators). These processes will be overseen by the Clinical Trials Unit to ensure protocol compliance as far as possible. Ambulance stations will be notified of activation with sufficient time to enable Valsalva pouches to be changed and all station clinicians to be notified. No other individuals outside the Lead Research Paramedic, logistics supply chain and the station clinicians will be made aware of the randomisation.

The timing and duration of cluster activation will only be changed in response to unexpected (high or low) rates of participant recruitment and only by agreement of the Trial Management Group (TMG), Trial Steering Committee (TSC) and sponsor.

To maintain blinding of research staff, all station clusters will be given a unique code that will be used in the clinical report form (CRF). A specific protocol (see below) will also be used to accurately identify the treating cluster (and hence treatment allocation) at the time of patient participation.

### EVADE cluster identification protocol

The process for determining the origin of the attending ambulance (and hence the equipment bag available to the treating clinician and allocation) will be according to a strict protocol. This will ascribe the correct cluster assignment while maintaining researcher blinding. Once the cluster is identified, it will only be identified in the CRF by its unique trial ID number. The hierarchy for determining cluster identification (and therefore, the intention-to-treat treatment allocation) is summarised below. Where more than one crew has attended a participant, it will be the first treating crew (as long as they are from a participating station) that will be used to identify the station allocation. The actual treatment used (as recorded in the ePCR) will also be recorded to inform any per-protocol analysis.

The treating cluster will be identified by:

1. Determining in the ePCR, which station the standard equipment bag originated from.
2. If the station/cluster cannot be confirmed in this way, the lead clinicians involved will be emailed (likely one of two possible stations).
3. Failing that, station champions will be emailed to request local inspection of drugs books (again likely to be one of two possible stations).
4. Finally, if the cluster cannot be identified through the above steps, support will be requested from the SWASFT Clinical Information and Records Office to access the computer assisted dispatch system to provide geographical location coordinates but not address to identify the most likely treating cluster. If required, the Global Rostering System may also be used to identify which ambulance stations clinicians were working from.

### Independent ECG review

To estimate diagnostic appropriateness, a review of available pre-VM ECGs from the ePCR will be undertaken by an independent assessor (consultant electrophysiologist) blind to treatment allocation. Traces with severe interference, preventing interpretation, will be excluded. All other traces will be classified as eligible for VM (eg, re-entrant SVT, undetermined SVT) or ineligible VM (eg, atrial fibrillation, atrial flutter, sinus tachycardia). In practice, for normal treating clinicians, it can sometimes be challenging to distinguish between some of these ECG rhythms and VM is appropriate and safe if clinicians believe, on the evidence available, that the patient has an eligible SVT.

### Safety monitoring

The VM is a very safe, standard intervention with no evidence of harm in previous large studies. There is no reason to believe the trial intervention will pose any additional risks or significant delay in the patient’s treatment. However, any problems or adverse events during any VM or in the care of trial patients will be recorded, closely monitored and reviewed by the TMG and TSC. Adverse events will be recorded by the prehospital clinician in the ‘Datix’ reporting system used by SWASFT, screened for during data entry and brought to the attention of the Lead Research Paramedic for further consideration. Further ambulance attendances over the following 3 months from the index attendance will also be reviewed and monitored as part of trial safety monitoring.

An adverse event is classified as a serious adverse event (SAE) if it results in death, is life-threatening, requires hospitalisation or prolongation of existing hospitalisation, results in persistent or significant disability or incapacity, causes a congenital anomaly or is considered by the investigator to be an important medical event. Admission to hospital required for the presenting SVT or associated condition will not be considered an SAE in this trial. However, the reason for admission will be recorded and any admissions that are not clearly related to the participant’s presenting condition will be reviewed to assess whether they should be recorded as an SAE.

Any SAE recorded through the Datix system will also be brought to the attention of the chief investigator, who will assess it for possible causality linked to the trial in any way. All SAEs will be reported to the sponsor and reviewed by the TSC. The trial may be stopped if any indication of harm is found.

### Outcome measures

#### Primary outcome

The primary outcome of the study will be the percentage of participants treated with a VM by the ambulance service, who are conveyed to hospital, as determined by the SWASFT ePCR. This includes a mandatory record of the participant’s final destination for each care episode. The reasons for conveyance will also be recorded.

#### Secondary outcomes

These will include the number of VM attempts used, evidence of postural modification, cardioversion (return to normal heart rhythm) as recorded by the treating ambulance clinician (including spontaneous cardioversion), total time of the ambulance episode and further ambulance attendances for SVT including subsequent conveyance to hospital (to rule out the possibility that treatment postpones attendance to hospital) during the 3-month period after the index presentation. Other outcome measures will include an estimate of diagnostic appropriateness (through the retrospective expert reading of available, interpretable pre-Valsalva ECG traces recorded in the ePCR) and health-related quality of life questionnaire (EQ-5D-5L) data collected from the optional online link, where available. Compliance with conveyance guidelines will be recorded and also, as part of the safety monitoring for the trial, ambulance attendance for any reason within 3 months of index presentation will be reported and monitored.

### Other measures

Confirmation of the Valsalva strain method used will be recorded. Demographic data, including ethnicity, will also be extracted from the ePCR to describe the extent to which the two groups (intervention vs control) are similar with respect to patient characteristics and that all patient groups, commensurate with the local population demographic, have the opportunity to take part in the research.

### Determination of sample size

The sample size was calculated using formulae presented by Hussey and Hughes.^17^ Assuming a baseline non-conveyance rate of 10% (from routine and feasibility study data)^8^ and a 10% point increase in non-conveyance with VAD, an intracluster correlation coefficient of 0.01 and 90% power at the two-sided 5% level of significance, we estimated that the 80 ambulance clusters will need to recruit a total of 800 participants (without repeat presentations) to the trial. The intracluster correlation coefficient was estimated to be 0.009 for non-conveyance for all reasons (not just SVT) using routine data from 94 SWASFT ambulance stations for care episodes for the 12 months between 1 February 2019 and 31 January 2020, inclusive.

Each ambulance cluster would be expected to recruit two participants in each of the five stages (each stage lasts 4 months) over the 20 months of recruitment. The target effect size was based on the minimum clinically meaningful effect that would justify a change in practice, taking into account the benefits and cost of the devices while remaining plausible according to the feasibility trial data (12% absolute increase in non-conveyance (95% CI: 3 to 34)).^8^ Prior to commencement of the trial, we also undertook a data collection exercise, using the proposed trial eligibility and data collection methods. This showed 63 (95% CI: 50 to 77) eligible patients had undergone a VM for SVT in SWASFT over 1 month. This number was in keeping with data from our feasibility study and previous service evaluations and in excess of that needed in our sample size estimates (40/month). This will allow for repeat presentations (around 10% of all cases by the end of an SVT VM trial of similar duration) and opt-out of data use, which, from our patient and public involvement and engagement and feasibility work, we expect to be low.

Due to the study design, recruitment will also largely be determined by normal practice rather than specific trial activities. It was felt that 20 months to recruit 800 participants is conservative and achievable. However, initial study progress criteria are proposed to monitor the recruitment rate (see table 2).

**Table 2:** Study progression criteria

Criteria	Green	Amber	Red
Individual-level recruitment	≥160 participants (100+%)	128–159 participants (80–99%)	<128 participants (below 80%)
Cluster-level recruitment	80 (100%) clusters each recruit at least one participant	72–79 clusters each recruit at least one participant (90–99%)	<72 clusters each recruit at least one participant (below 90%)

Progression criteria after 4 months of participant trial recruitment.

#### Progress (RAG) criteria

The stepped wedge design is predicated on reliably recruiting the number of participants specified by the sample size calculation at each stage of the trial. Monthly recruitment will be monitored closely by the TMG and regularly reported to the TSC. Recruitment below target (amber or red criteria) will trigger a remedial plan by the TMG with promotion of the SVT guideline and trial awareness and feedback on missed cases (where a VM was indicated but not undertaken) in addition to the planned promotion of the trial (newsletters, posters, etc) and NIHR CRN support. Financial constraints would preclude significant trial prolongation and if, despite these measures, trial recruitment was less than 80% of our overall target for the first 4 months of the study, we propose discussion with the TMG, TSC and funder to consider trial termination or agreed modification.

### Data analysis

Analysis will be conducted according to a prespecified statistical analysis plan written by the trial statisticians, approved by an independent statistician on the TSC and made publicly available ahead of database lock.

Participant and cluster (ambulance stations) characteristics will be summarised using means and SD or medians and IQRs for continuous variables and numbers and percentages for categorical variables, by condition status (intervention vs control) and overall.

In the main analyses, outcomes will be analysed using the intention-to-treat principle, based on intervention status of the cluster at the time of the VM, irrespective of whether there was evidence of device use. Per-protocol analyses will also be undertaken. Repeat presentations of patients previously recruited will be excluded from the primary analyses but included in secondary analyses. The intervention effect will be estimated through fitting mixed effects (‘multilevel’) regression models that allow for the correlation between outcomes of patients from the same cluster (station). The models will include fixed effects for the intervention effect and time (stage) and a random effect for the cluster.

Given the nature of the primary outcome and normally well-recorded routine data to be used in the trial, it is not anticipated that there will be a significant quantity of missing data. The trial Research Paramedic will make every attempt to retrieve any missing data. Should any key outcome data remain missing, sensitivity analyses may be proposed, using a range of assumptions where necessary and consideration given to suitable imputation.

### Data monitoring

All data will be managed according to the trial data management plan. Although no formal data monitoring committee is proposed, overall conveyance rates and adverse event data will be monitored by the TSC to ensure appropriateness and safety. An independent subgroup of the TSC will also assess unblinded conveyance and safety data by group allocation.

### Patient and public involvement

In line with INVOLVE guidelines,^18^ we have engaged patients at every stage of the study’s design. Our project plan has been discussed widely with three different patient and public groups including our feasibility study patient group, ambulance service patient representatives and the Exeter 10 000 (a volunteer group who review trial proposals) and adapted accordingly. There was wide support for our design which was felt to be appropriate, proportionate and respectful of patient views.

## Ethics and dissemination

The study was given regional ethics committee and Health Research Authority approval on 29 April 2022 (22/SC/0032) and complies with the Declaration of Helsinki and Good Clinical Practice guidelines.

The study results will be applicable and of interest to paramedics, ambulance trusts, emergency physicians, acute physicians, general practitioners and patients. We aim to disseminate the study findings to these groups through publication of the trial in appropriate international peer-reviewed journals according to the trial’s publication plan. Results will also be presented at appropriate local, national and international academic meetings such as the College of Emergency Medicine scientific meeting and International Conference of Emergency Medicine, as well as the 999 Emergency Medical Services Research Forum and through appropriate press releases and social media.

Patients recruited into the trial will have the opportunity to see the study’s findings through the website of the Arrhythmia Alliance, a heart rhythm charity and one of our collaborators, who have offered to post the trial results. Details of this organisation are included in the trial PIL. We will also present the trial on a publicly available study webpage^19^ of the SWASFT website.

## Discussion

The Evaluation of the prehospital use of a VAD in the Emergency treatment of SVT (EVADE SVT) trial is the first study to assess whether the routine availability of a simple device (VAD) and its instructions to assist the ambulance service delivery of the VM to treat SVT reduces the proportion of patients with SVT conveyed to hospital. In light of detailed feasibility work and patient advice, the study has been specifically designed to be delivered during routine care by normal clinical staff within the prehospital setting.

The proposed study is powered to detect a meaningful difference in conveyance rate which would have important benefits for patients and emergency services. Successfully treated patients will feel better sooner and avoid disruption to their lives and potentially long waits and further treatment in the emergency department. If a reduction in conveyance rate is demonstrated when the VAD is made available, the reduced need for patients to be brought to emergency departments would also deliver potential savings for ambulance services that would cover the cost of these devices within 7 months and provide patients with an easy-to-use device to keep for self-treatment. Frontline ambulances would also be available sooner to attend other emergencies and emergency departments would have fewer patients to assess and treat.

An innovative trial design (in this setting and challenging environment for research) has been proposed to deliver the aims of the project, while maintaining scientific integrity and at a cost commensurate with the condition. The stepped wedge design will enable the randomised introduction of VAD availability as standard care. This ensures patients receive timely emergency care by normal treating prehospital clinicians, without additional research burdens, while still giving patients the opportunity to contribute to research should they wish to be included. A robust system to ensure patients’ prior wishes regarding use of routine clinical data for research are respected and that all patients can opt out should they not wish to take part, ensures that Good Clinical Practice principles are maintained. Importantly, this approach was driven and supported by the project’s public and patient engagement work.

The use of routine data and delivery of the trial during normal care will contribute to study efficiency and help to ensure that study findings will be representative of real-life practice and improve external validity. Service-wide introduction of the intervention during normal practice, with assessment of the overall device use across the trust, will also give some measure of its acceptability and ease of implementation. This will also be useful to other ambulance trusts when considering the study findings.

The primary outcome of the study was carefully considered. Cardioversion was contemplated as an alternative primary outcome, but our feasibility study and other previous research have demonstrated challenges in providing robust evidence and timing of cardioversion. Conveyance is a clear, measurable outcome which is important to patients, clinicians and health services.

The study design means the trial recruitment rate will also be determined by normal practice. While this will ensure all eligible adult patients undergoing an SWASFT-delivered VM can be included in the trial, recruitment could potentially be affected by other factors affecting ambulance availability and wider pressures on the ambulance service. Previous feasibility work demonstrated a consistent incidence of VM treatment, at various time points, including during the COVID-19 pandemic. These data were used to determine the anticipated duration of the stepped wedge phases and overall trial duration. However, there is no certainty that the previously observed incidence rate will be maintained throughout this definitive trial. Recent, well-documented ambulance delays could affect recruitment rates if, for example, more patients make their own way to hospital.

The planned analysis of health-related quality of life (EQ-5D-5L) data through an optional questionnaire will be dependent on response rates to an invitation included in the PIL. Although it is known that response rates to health questionnaires are notoriously low in emergency settings,^20^ the main trial design and resources for the study do not allow for an alternative approach to collect these data. Recruitment and EQ-5D response rates will therefore be closely monitored and action considered in conjunction with the trial oversight committee, sponsor and funder, as required.

## Conclusions

The EVADE SVT trial will seek to determine whether the routine availability of a simple device (VAD) and its instructions to assist the ambulance service delivery of the VM to treat SVT reduces the proportion of patients with SVT that are conveyed to hospital. This trial protocol has been developed to deliver this trial within one large UK ambulance service during routine care. It is designed to detect a meaningful change in conveyance rate that would be of benefit to patients and emergency services and be relevant to other ambulance and emergency services around the world.

### Protocol version

This paper is based upon the EVADE SVT study final protocol V.2.1, dated 18 July 2022.

## Supplementary Material

Reviewer comments

Author's manuscript
