# Supplementary material for: Evaluation of the prehospital use of a Valsalva assist device in the emergency treatment of supraventricular tachycardia (EVADE SVT): study protocol for a stepped wedge cluster randomised controlled trial
Source: BMJ Open. 2023 Jun 8;13(6):e073315. doi: 10.1136/bmjopen-2023-073315 (PMC10255124; doi:10.1136/bmjopen-2023-073315)
Supplement: Author's manuscript [file bmjopen-2023-073315.draft_revisions.pdf]

BMJ Open is committed to open peer review. As part of this commitment we make the peer review history of every article we publish publicly available.

When an article is published we post the peer reviewers' comments and the authors' responses online. We also post the versions of the paper that were used during peer review. These are the versions that the peer review comments apply to.

The versions of the paper that follow are the versions that were submitted during the peer review process. They are not the versions of record or the final published versions. They should not be cited or distributed as the published version of this manuscript.

BMJ Open is an open access journal and the full, final, typeset and author-corrected version of record of the manuscript is available on our site with no access controls, subscription charges or pay-per-view fees (http://bmjopen.bmj.com).

If you have any questions on BMJ Open's open peer review process please email info.bmjopen@bmj.com

BMJ Open

Evaluation of the pre-hospital use of a Valsalva assist device in the emergency treatment of supraventricular tachycardia (EVADE SVT): study protocol for a stepped wedge cluster randomised controlled trial

Journal:	*BMJ Open*
Manuscript ID	bmjopen-2023-073315
Article Type:	Protocol
Date Submitted by the Author:	06-Mar-2023

Complete List of Authors:	Appelboam, Andrew; Royal Devon University Healthcare NHS Foundation Trust, Emergency Department<br>Osborne, Ria; South Western Ambulance Service NHS Foundation Trust<br>Ukoumunne, Obioha; University of Exeter, NIHR CLAHRC South West Peninsula (PenCLAHRC)<br>Black, Sarah; South Western Ambulance Service NHS Foundation Trust, Research, Audit and Improvement<br>Boot, Suzanne; South Western Ambulance Service NHS Foundation Trust<br>Richards, Nicolas; South Western Ambulance Service NHS Foundation Trust<br>Scotney, Natalie; South Western Ambulance Service NHS Foundation Trust<br>Rhodes, Shelley; University of Exeter, Medical School<br>Cranston, Tim; University of Exeter, Medical School<br>Hawker, Ruth; South Western Ambulance Service NHS Foundation Trust, PPI Representative, honorary SWASFT contract.<br>Gillett, Annette; University of Exeter Medical School, Peninsula Childhood Disability Research Unit (PenCRU)<br>Jones, Ben; University of Exeter, Medical statistics<br>Hawton, Annie; Peninsula Medical School<br>Dayer, Mark; Taunton and Somerset NHS Foundation Trust, Cardiology<br>Creanor, Siobhan; University of Exeter<br>EVADE SVT study collaborators, EVADE SVT study collaborators; South Western Ambulance Service NHS Foundation Trust

Keywords:	ACCIDENT & EMERGENCY MEDICINE, Pacing & electrophysiology < CARDIOLOGY, Adult cardiology < CARDIOLOGY

SCHOLARONE™
Manuscripts

Evaluation of the pre-hospital use of a Valsalva assist device in the emergency treatment of supraventricular tachycardia (EVADE SVT): study protocol for a stepped wedge cluster randomised controlled trial

Andrew Appelboam[1], Ria Osborne[2], Obioha C Ukoumunne[3], Sarah Black[2], Suzanne Boot[2], Nicolas Richards[2], Natalie Scotney[2], Shelley Rhodes[4], Tim Cranston[4], Ruth Hawker[5], Annette Gillet[3], Ben Jones[3], Annie Hawton[6], Mark Dayer[7], Siobhan Creanor[4] and the EVADE SVT study collaborators*

[1]Academic Department of Emergency Medicine, Royal Devon University Healthcare NHS Foundation Trust, Exeter; [2]South Western Ambulance Service NHS Foundation Trust; [3]NIHR Applied Research Collaboration South West Peninsula, Department of Health and Community Sciences, Faculty of Health and Life Sciences, University of Exeter; [4]Exeter Clinical Trials Unit, University of Exeter; [5]Patient and public representative; [6]Health Economics Group, University of Exeter, [7]Department of Cardiology, Somerset NHS Foundation Trust.; University of Plymouth, Faculty of Health

Corresponding Author: Andrew Appelboam.

c/o Emergency Department, Royal Devon University Healthcare NHS Foundation Trust, Barrack Road, Exeter, Devon EX2 3DW

Tel : 00 44 (0)1392 402170

Fax : 00 44 (0)1392 402313

Email: EVADE@SWAST.nhs.uk

Trial Sponsor : South Western Ambulance Service NHS Foundation Trust, Abbey Court, Eagle Way, Exeter, EX2 7HY

Keywords : Supraventricular tachycardia, Valsalva manouvre, emergency care

For peer review only - http://bmjopen.bmj.com/site/about/guidelines.xhtml

Abstract

Introduction: Patients with episodes of supraventricular tachycardia (SVT), a common heart arrhythmia, are often attended by ambulance services. International guidelines advocate treatment with the Valsalva Manoeuvre (VM), but this simple physical treatment has a low success rate, with most patients requiring conveyance to hospital. The Valsalva Assist Device (VAD) is a simple device that might help practitioners and patients perform a more effective VM and reduce the need for patients to be taken to hospital.

Methods and analysis: This stepped wedge cluster randomised controlled trial, conducted within a UK ambulance service, compares the current standard VM with a VAD-delivered VM in stable adult patients presenting to the ambulance service with SVT. The primary outcome is conveyance to hospital; secondary outcomes measures include cardioversion rates, duration of ambulance care and number of subsequent episodes of SVT requiring ambulance service care. We plan to recruit approximately 800 patients, to have 90% power to detect an absolute reduction in conveyance rate of 10% (from 90% to 80%) between the standard VM (control) and VAD-delivered VM (intervention). Such a reduction in conveyance would benefit patients, the ambulance service and receiving emergency departments. It is estimated potential savings would pay for devices for the entire ambulance trust within seven months.

Ethics and dissemination: The study has been approved by the Oxford Research Ethics Committee (reference 22/SC/0032). Dissemination will be through peer-reviewed journal publication, presentation at national and international conferences and by the Arrhythmia Alliance, a patient support charity.

Registration: Current Controlled Trials (ISRCTN 16145266). Adopted by the National Institute for Health Research (NIHR) Clinical Research Network.

Abstract word count: 264

For peer review only - http://bmjopen.bmj.com/site/about/guidelines.xhtml

Article Summary

Strengths and limitations of this study

- This is the first randomised controlled trial to assess the potential benefits of a bespoke Valsalva Assist Device to deliver the Valsalva Manoeuvre in the treatment of patients presenting with supraventricular tachycardia (SVT) to an ambulance service in the UK.
- This efficient, pragmatic, stepped wedge trial is designed to be delivered during normal ambulance service care of patients with SVT and use routinely collected data to assess for a meaningful reduction in the rate of conveyance to hospital, as the device is brought into standard use.
- Although individual participant randomisation and blinding is not possible in this study, blinding of the trial statisticians will be maintained until the statistical analysis plan is finalised and signed off by the Trial Steering Committee.
- The study is designed to detect a difference in conveyance rates that would be meaningful to patients, the ambulance service and receiving hospitals and of a size and affordability to encourage clinicians and ambulance services to stock and use the device.

For peer review only

For peer review only - http://bmjopen.bmj.com/site/about/guidelines.xhtml

Introduction

Supraventricular tachycardias (SVT) are a common heart rhythm disorder causing episodes of fast heart rates.. The incidence of SVT is estimated to be 35 episodes per 100,000 persons/year[1] and attacks are unpleasant and disruptive to patients' lives[2]. SVT often occurs because of an additional electrical pathway in the heart, causing a 're-entrant tachycardia'.

Pre-hospital emergency services often attend patients with attacks of SVT. A regional Australian ambulance service recorded seeing 800 cases of SVT in a year[3] with audit data suggesting a similar number of episodes in the South Western Ambulance Service NHS Foundation Trust (SWASFT), in the South West of England, UK. International ambulance treatment guidelines recommend initial treatment with the Valsalva Manoeuvre (VM)[4,5]. This simple physical treatment involves an exhalation strain against resistance which causes a slowing of the heart (mediated by the vagal nerve) and can cardiovert an SVT back to normal sinus rhythm. Unfortunately, cardioversion rates in routine pre-hospital practice can be as low as 5% and most patients are conveyed to hospital with ongoing SVT[6,7].

Results from our feasibility study suggested SWASFT ambulance clinicians treat around 500 of the SVT episodes they see each year, using a VM with approximately 90% of these patients being conveyed to hospital[8]. This ambulance service has previously adopted the modified Valsalva manoeuvre (mVM) into its guidelines as the first-line treatment in stable patients with SVT. This guideline, in common with others, recommends that the VM strain is delivered by blowing into a 10 ml syringe[9] followed by an internationally recommended[10] postural modification which has shown to improve cardioversion rates[11]. However, syringes are unreliable in generating the correct VM strain pressure[12] and remembering the modified manoeuvre may be difficult for individual clinicians, who only occasionally see these patients.

A simple, easy to use Valsalva Assist Device (VAD) has been developed which provides the correct, recommended resistance (40mmHg) for the VM exhalation strain.[13] A CE-marked VAD has been produced by Meditech Systems Limited (www.meditechsystems.co.uk), marketed as the 'Valsa-Valve' Valsalva Assist Device (see figure 1). This VAD includes instructions for use on the device and it's packaging, including information on the timings and the postural changes required for the mVM.

For peer review only - http://bmjopen.bmj.com/site/about/guidelines.xhtml

If appropriate, these single-patient-use devices can also be left with the patient, for subsequent use should an attack recur. The VAD might improve the quality of the VM and increase the chances of cardioversion and non-conveyance compared to the standard approach using syringe delivered VMs. Reducing ambulance conveyance of patients to hospital, when appropriate and possible, is increasingly important to health services and patients[14].

A definitive randomised controlled trial is needed to test whether the availability of a VAD to ambulance clinicians, when treating patients with SVT, affects the success of treatment and the need for conveyance to hospital, compared to current practice. A recent feasibility study and patient and public feedback have informed the nature and design of the definitive trial outlined in this protocol paper.

For peer review only

Methods and analysis

General study design and conduct:

This study is an efficient, pragmatic, stepped wedge cluster randomised controlled trial to see if the routine availability of a VAD and its instructions (intervention), to treat adult patients presenting to SWASFT ambulance clinicians with SVT, can reduce the rate of hospital conveyance compared with standard practice (control). The proposed stepped wedge design involves the allocation of ambulance service clusters across the whole service. These clusters all start as controls, providing current standard care, before being randomly assigned to using the VAD, as a new standard treatment, in defined groups and periods over the duration of the study (further details provided below). This study protocol is designed and reported according to SPIRIT reporting guidelines.[15]

Study population and setting:

Eligible patients presenting to SWASFT with suspected SVT and who are treated with a VM by a SWASFT ambulance clinician, will be included. The study will be conducted entirely within SWASFT, UK. This ambulance service uses a searchable electronic patient care record (ePCR) as standard when attending patients. This was shown to be a reliable and easily searchable data source in our feasibility study. SWASFT also has an established clinical guideline advocating the use of a modified VM and non-conveyance of successfully treated SVT patients.

For peer review only - http://bmjopen.bmj.com/site/about/guidelines.xhtml

The study aims to include all adult patients aged 18 years and over for whom there is a record of a Valsalva Manoeuvre delivered by an ambulance clinician, working out of a participating ambulance station, for presumed SVT.

Patients who subsequently withdraw consent for use of their data or have an existing general opt-out for use of routine NHS data for research will be excluded from the trial. Data from patients who are prisoners at the time of treatment, patients with permanent lack of capacity as recorded by the treating clinician and patients solely treated by a SWASFT member of staff outside of the included ambulance station clusters (e.g., Air Ambulance, Hazardous Area Response Team, British Association for Immediate Care (BASICS) Doctor) will also be excluded. Data from patients previously included in the study will be excluded from the primary outcome analysis but will be used in analyses of some secondary outcomes. A summary table of the trial eligibility criteria is shown in Table 1.

Table 1: Trial Inclusion and Exclusion Criteria

Inclusion criteria	• Adult patients (age 18 years or over)<br>• Record of a Valsalva manoeuvre for presumed SVT having been delivered by an ambulance clinician asigned to a participating ambulance station cluster
Exclusion criteria	• Subsequent withdrawal of patient consent to use their data<br>• Existing opt-out for use of NHS data for research<br>• Data from patients who are prisoners at the time of treatment<br>• Patients with permanent lack of capacity as recorded by the treating clinician<br>• Patients solely treated by a SWASFT resource not from one of the included ambulance station clusters e.g. Air Ambulance, Hazardous Area Response Team,Tiverton Minor Injuries Unit, British Association for Immediate Care (BASICS) Doctor<br>• Data from patients previously included in the study will be excluded from the primary outcome analysis but will be used in analyses of some secondary outcomes

For peer review only - http://bmjopen.bmj.com/site/about/guidelines.xhtml

Study procedures:

Potential study participants will undergo standard clinical assessment by ambulance clinicians. For suspected SVT this typically includes medical history, recording of routine initial observations of pulse, blood pressure, respiratory rate, oxygen saturation and the recording of a 12-lead electrocardiogram (ECG). Routinely collected clinical data are entered into the ePCR system and so are available for subsequent trial data extraction for eligible participants.

The screening of eligibility for VM treatment, the conduct of the VM and all other aspects of clinical care will be entirely governed by SWASFT clinical guidelines, interpreted and followed at the discretion of the treating clinician. On commencement of the intervention phase of the trial, SWASFT clinical guidelines will be updated to include the use of the VAD, where available as part of standard care.

Trial treatment:

The stocking of ambulances with the VAD (and thus the allocation of interventions) will be determined by the randomisation schedule as described below. This will ensure that a VAD is available for use by the treating clinician according to the randomisation schedule. The use of devices will thereafter be at the discretion of the treating clinician but encouraged through trial training and publicity; device use will be recorded and used as a measure of device acceptability.

Post-treatment assessment usually includes the recording of a further 12-lead ECG to determine whether normal sinus rhythm has been restored. However, this is not universal and often omitted if there has been no clear change in the heart rate and rhythm. SWASFT guidelines allow for non-conveyance in stable, well patients who have cardioverted, have normal observations and remain in sinus rhythm after 20 minutes of observation. In this case, patients are advised to seek further follow-up with their general practitioner. Patients will also be given the device (syringe or VAD) they used to perform the VM to take away, with written instructions, in case of recurrent SVT attacks.

Consent:

All participants will be given a participant information leaflet (PIL), specific to the stage of the trial, at a convenient time after treatment, at the discretion of the treating

For peer review only - http://bmjopen.bmj.com/site/about/guidelines.xhtml

ambulance clinician. The front of the PIL will clearly state that the information can be read when the patient is ready and feels well enough. The PIL contains an explanation of SVT, the nature of the trial and how to opt out, should they not want their data to be used. Ambulance clinicians will be asked to record in the ePCR, confirmation that the PIL was given to the patient or a carer. As part of the screening and data entry process, the Lead Research Paramedic will confirm whether a PIL was given by checking the ePCR and, if not recorded, will contact the treating paramedic to confirm. If there is no evidence that a PIL was given at the time of treatment, the Lead Research Paramedic will post a PIL to the patient.

For patients with temporary lack of capacity (for example due to their condition or associated illness) the PIL will be given to the next of kin or carer, if available, to explain and share with the patient when they are well enough. Patients with permanent severe lack of capacity, as determined by the treating ambulance clinician, will not be included. PIL translations will be sent to non-English speakers by SWASFT research staff where possible.

The PIL gives details of how patients can opt out of their routine data being used in the trial. This can be through immediate verbal opt-out (to the treating ambulance clinician) or retrospectively by telephone, post or email. Immediate verbal opt-outs will be recorded on the ePCR and screening of cases for inclusion in the trial dataset will include a full search for opt-outs in the ePCR or received by any other means. An opt-out received at any time during the trial will render the patient ineligible, and their clinical data for their initial and subsequent episodes will not be included in the study. Safety follow-up data, already recorded up to the point of withdrawal, will be kept.

In the UK, patients also have the option to nationally opt out of having any of their routine digital NHS data used for research. The national rate of this type of opt-out currently stands at around 5%. After taking advice from the regional research ethics committee and our public and patient advisory group, we have elected to exclude all patients with a national data opt out unless the patient advises that they want to be included in the study (details of how to do this are included in the PIL).

In the absence of (i) the patient having a national opt-out and (ii) the research team receiving any individual opt-out, inferred consent for routine data use will be assumed.

For peer review only - http://bmjopen.bmj.com/site/about/guidelines.xhtml

There is a precedent for this approach to collecting routine data for emergency medicine research and it is considered to constitute active recruitment as stated in the NIHR Clinical Research Network (CRN) recruitment policy document[16].

Individual trial participation will last from the start of the SVT ambulance service care episode until three months after this time to enable the collection of further ambulance service use data as part of the planned recurrent SVT and safety follow-up period.

Health-related quality of life data:

The PIL also includes an invitation to an optional questionnaire to collect (non-routine) data on the effects of the participant's condition and ambulance service treatment on their health-related quality of life. The invitation includes a web link (with a QR code) to this part of the study, printed on the PIL. Those who choose to participate will be given further information and asked for consent if they follow this link. Once they have given consent, they will be asked to complete the EQ-5D-5L questionnaire online, ideally within a week of the patient feeling well enough to complete the form; this will also be explained in the PIL. Participants who consent to this study component will be contacted digitally and re-invited to complete the EQ-5D-5L, three months after the SVT ambulance service care episode. Data from completed forms will be linked with the study database entry for the index presentation. Paper or emailed copies of the EQ-5D-5L will be sent to participants if requested, as outlined in the PIL. Paper or online EQ-5D-5L will be used under licence.

Formation of ambulance station clusters and randomisation:

Ambulance station clusters will be formed from the 93 SWASFT ambulance stations in existence at the time of study setup. Recent historic data on activity and general conveyance rates for all stations were collected to inform the formation of trial clusters and sample size calculations. After merging very small and closely located stations, whilst maintaining the largest possible number of similar sized clusters, 80 (mostly single station) ambulance station clusters were identified. Clinicians working from these stations use equipment bags strictly belonging to and stocked by that station.

The stepped wedge trial design is comprised of five stages, with each stage lasting four months. In the first stage, none of the station clusters will have access to the VAD and patients will be treated as per standard practice. The clusters will then be randomly

For peer review only - http://bmjopen.bmj.com/site/about/guidelines.xhtml

allocated, by computer-generated sequences (stratified by location and balanced on cluster size), to include VADs with their equipment bags, in one of the 4 remaining stages, so that 20 clusters and their clinicians receive access to the VAD for the first time at each stage. Randomisation will be performed by an independent statistician otherwise unconnected with the trial.

Once the VAD has been introduced, the cluster will retain the use of devices for the remainder of the study so that by the fifth stage, all 80 clusters and their clinicians will have access to the VAD (see Figure 2). Devices will be added to a clearly marked 'Valsalva pouch' attached to the standard equipment bag. These bags are checked and re-stocked daily and carried by all treating ambulances. All station clusters randomised to VAD use will initiate availability of the VAD on the same day, coordinated by SWASFT supply logistics and the local ambulance station 'champions' (listed as trial collaborators). These processes will be overseen by the Clinical Trials Unit (CTU) to ensure protocol compliance as far as possible. Ambulance stations will be notified of activation with sufficient time to enable Valsalva pouches to be changed and all station clinicians to be notified. No other individuals outside the Lead Research Paramedic, logistics supply chain and the station clinicians will be made aware of the randomisation.

The timing and duration of cluster activation will only be changed in response to unexpected (high or low) rates of participant recruitment and only by agreement of the Trial Management Group (TMG), Trial Steering Committee (TSC) and sponsor.

For peer review only

To maintain blinding of research staff, all station clusters will be given a unique code that will used in the clinical report form (CRF). A specific protocol (see below) will also be used to accurately identify the treating cluster (and hence treatment allocation) at the time of patient participation.

EVADE Cluster Identification Protocol:

The process for determining the origin of the attending ambulance (and hence the equipment bag available to the treating clinician and allocation) will be according to a strict protocol. This will ascribe the correct cluster assignment whilst maintaining researcher blinding. Once the cluster is identified, it will only be identified in the CRF by its unique trial ID number. The hierarchy for determining cluster identification (and therefore, the intention-to-treat treatment allocation) is summarised below. Where more than one crew have attended a participant, it will be the first treating crew (as long as they are from a participating station) that will be used to identify the station allocation. The actual treatment used (as recorded in the ePCR) will also be recorded to inform any per protocol analysis.

The treating cluster will be identified by:

1. Determining in the ePCR, which station the standard equipment bag originated from.
2. If the station / cluster cannot be confirmed in this way, the lead clinicians involved will be emailed (likely one of two possible stations).
3. Failing that, station champions will be emailed to request local inspection of drugs books (again likely to be one of two possible stations).
4. Finally, if the cluster cannot be identified through the above steps, support will be requested from the SWASFT Clinical Information and Records Office to access the computer assisted dispatch (CAD) system to provide geographical location coordinates but not address to identify the most likely treating cluster. If required, the Global Rostering System (GRS) may also be used to identify which ambulance stations clinicians were working from.

For peer review only - http://bmjopen.bmj.com/site/about/guidelines.xhtml

Independent ECG review:

To estimate diagnostic appropriateness, a review of available pre-VM ECGs from the ePCR will be undertaken by an independent assessor (consultant electrophysiologist) blind to treatment allocation. Traces with severe interference, preventing interpretation, will be excluded. All other traces will be classified as eligible for VM (e.g., re-entrant SVT, undetermined SVT) or ineligible VM (e.g., atrial fibrillation, atrial flutter, sinus tachycardia). In practice, for normal treating clinicians, it can sometimes be challenging to distinguish between some of these ECG rhythms and VM is appropriate and safe if clinicians believe, on the evidence available, that the patient has an eligible SVT.

Safety monitoring:

The VM is a very safe, standard intervention with no evidence of harm in previous large studies. There is no reason to believe the trial intervention will pose any additional risks or significant delay in the patient's treatment. However, any problems or adverse events during any VM or in the care of trial patients will be recorded, closely monitored and reviewed by the TMG and TSC. Adverse events will be recorded by the pre-hospital clinician in the 'Datix' reporting system used by SWASFT, screened for during data entry and brought to the attention of the Lead Research Paramedic for further consideration. Further ambulance attendances over the following three months from the index attendance will also be reviewed and monitored as part of trial safety monitoring.

An adverse event is classified as a Serious Adverse Event (SAE) if it results in death, is life-threatening, requires hospitalisation or prolongation of existing hospitalisation, results in persistent or significant disability or incapacity, causes a congenital anomaly or is considered by the investigator to be an important medical event. Admission to hospital required for the presenting SVT or associated condition will not be considered an SAE in this trial. However, the reason for admission will be recorded and any admissions that are not clearly related to the participant's presenting condition will be reviewed to assess whether they should be recorded as an SAE.

Any SAE recorded through the Datix system will also be brought to the attention of the Chief Investigator, who will assess it for possible causality linked to the trial in any way.

For peer review only - http://bmjopen.bmj.com/site/about/guidelines.xhtml

For peer review only

All SAEs will be reported to the sponsor and reviewed by the TSC. The trial may be stopped if any indication of harm is found.

Outcome measures:

Primary Outcome: The primary outcome of the study will be the percentage of participants treated with a Valsalva manoeuvre (VM) by the ambulance service, who are conveyed to hospital, as determined by the SWASFT electronic patient care record (ePCR). This includes a mandatory record of the participant's final destination for each care episode. The reasons for conveyance will also be recorded.

Secondary Outcomes: These will include the number of VM attempts used, evidence of postural modification, cardioversion (return to normal heart rhythm) as recorded by the treating ambulance clinician (including spontaneous cardioversion), total time of the ambulance episode and further ambulance attendances for SVT including subsequent conveyance to hospital (to rule out the possibility that treatment postpones attendance to hospital) during the three month period after the index presentation. Other outcome measures will include an estimate of diagnostic appropriateness (through the retrospective expert reading of available, interpretable pre-Valsalva ECG traces recorded in the ePCR) and health-related quality of life questionnaire (EQ-5D-5L) data collected from the optional online link, where available. Compliance with conveyance guidelines will be recorded and also, as part of the safety monitoring for the trial, ambulance attendance for any reason within three months of index presentation will be reported and monitored.

Other measures

Confirmation of the Valsalva strain method used will be recorded. Demographic data, including ethnicity, will also be extracted from the ePCR to describe the extent to which the two groups (intervention versus control) are similar with respect to patient characteristics and that all patient groups, commensurate with the local population demographic, have the opportunity to take part in the research.

Determination of sample size:

The sample size was calculated using formulae presented by Hussey and Hughes [17]. Assuming a baseline non-conveyance rate of 10% (from routine and feasibility study data[8]) and a 10% point increase in non-conveyance with VAD, an intra-cluster

For peer review only - http://bmjopen.bmj.com/site/about/guidelines.xhtml

correlation coefficient of 0.01 and 90% power at the two-sided 5% level of significance, we estimated that the 80 ambulance clusters will need to recruit a total of 800 participants (without repeat presentations) to the trial. The intra-cluster correlation coefficient was estimated to be 0.009 for non-conveyance for all reasons (not just supraventricular tachycardia) using routine data from 94 SWASFT ambulance stations for care episodes for the 12 months between 1st February 2019 and 31st January 2020, inclusive.

Each ambulance cluster would be expected to recruit two participants in each of the five stages (each stage lasts four months) over the 20 months of recruitment. The target effect size was based on the minimum clinically meaningful effect that would justify a change in practice, taking into account the benefits and cost of the devices whilst remaining plausible according to the feasibility trial data (12% absolute increase in non-conveyance (95% confidence interval (CI): 3-34[8])). Prior to commencement of the trial, we also undertook a data collection exercise, using the proposed trial eligibility and data collection methods. This showed 63 (95% CI: 50-77) eligible patients had undergone a VM for SVT in SWASFT over one month. This number was in keeping with data from our feasibility study and previous service evaluations and in excess of that needed in our sample size estimates (40/month). This will allow for repeat presentations (around 10% of all cases by the end of an SVT VM trial of similar duration) and opt-out of data use, which, from our Patient and Public Involvement and Engagement (PPIE) and feasibility work, we expect to be low.

Due to the study design, recruitment will also largely be determined by normal practice rather than specific trial activities. It was felt that 20 months to recruit 800 participants is conservative and achievable. However, initial study progress criteria are proposed to monitor the recruitment rate (see Table 2).

Progress (RAG) Criteria: The stepped wedge design is predicated on reliably recruiting the number of participants specified by the sample size calculation at each stage of the trial. Monthly recruitment will be monitored closely by the TMG and regularly reported to the TSC. Recruitment below target (amber or red criteria) will trigger a remedial plan by the TMG with promotion of the SVT guideline and trial awareness and feedback on missed cases (where a VM was indicated but not undertaken) in addition to the planned promotion of the trial (newsletters, posters, etc.) and NIHR

For peer review only - http://bmjopen.bmj.com/site/about/guidelines.xhtml

CRN support. Financial constraints would preclude significant trial prolongation and if, despite these measures, trial recruitment was less than 80% of our overall target for the first four months of the study, we propose discussion with the TMG, TSC and funder to consider trial termination or agreed modification.

Table 2: Study Progression Criteria

Progression Criteria At 4 Months

Criteria	**Green**	**Amber**	**Red**
Individual-level recruitment	≥ 160 participants (100+%)	128 to 159 participants (80-99%)	< 128 participants (below 80%)
Cluster-level recruitment	80 (100%) clusters each recruit at least one participant	72 to 79 clusters each recruit at least one participant (90-99%)	< 72 clusters each recruit at least one participant (below 90%)

Data analysis:

Analysis will be conducted according to a pre-specified statistical analysis plan (SAP) written by the trial statisticians, approved by an independent statistician on the trial steering committee and made publicly available ahead of database lock.

Participant and cluster (ambulance stations) characteristics will be summarised using means and standard deviations or medians and inter-quartile ranges for continuous variables and numbers and percentages for categorical variables, by condition status (intervention versus control) and overall.

In the main analyses, outcomes will be analysed using the intention-to-treat principle, based on intervention status of the cluster at the time of the VM, irrespective of whether there was evidence of device use. Per protocol analyses will also be undertaken. Repeat presentations of patients previously recruited will be excluded from the primary analyses but included in secondary analyses. The intervention effect will be estimated through fitting mixed effects (“multilevel”) regression models that allow for the correlation between outcomes of patients from the same cluster (station). The models will include fixed effects for the intervention effect and time (stage) and a random effect for the cluster.

For peer review only

For peer review only - http://bmjopen.bmj.com/site/about/guidelines.xhtml

Given the nature of the primary outcome and normally well-recorded routine data to be used in the trial, it is not anticipated that there will be a significant quantity of missing data. The trial Research Paramedic will make every attempt to retrieve any missing data. Should any key outcome data remain missing, sensitivity analyses may be proposed, using a range of assumptions where necessary, and consideration given to suitable imputation.

Data monitoring:

All data will be managed according to the trial data management plan. Although no formal data monitoring committee is proposed, overall conveyance rates and adverse event data will be monitored by the TSC to ensure appropriateness and safety. An independent sub-group of the TSC will also assess unblinded conveyance and safety data by group allocation.

Ethics and dissemination

The study was given Regional ethics committee (REC) and Health Research Authority (HRA) approval on 29th April 2022 (22/SC/0032) and complies with the Declaration of Helsinki and GCP guidelines.

The study results will be applicable and of interest to paramedics, ambulance trusts, emergency physicians, acute physicians, general practitioners and patients. We aim to disseminate the study findings to these groups through publication of the trial in appropriate international peer-reviewed journals according to the trial's publication plan. Results will also be presented at appropriate local, national and international academic meetings such as the College of Emergency Medicine scientific meeting and International Conference of Emergency Medicine, as well as the 999 Emergency Medical Services Research Forum and through appropriate press releases and social media.

Patients recruited into the trial will have the opportunity to see the study's findings through the website of the Arrhythmia Alliance, a heart rhythm charity and one of our collaborators, who have offered to post the trial results. Details of this organisation are included in the trial PIL. We will also present the trial on a publicly available study webpage[18] of the SWASFT website.

For peer review only - http://bmjopen.bmj.com/site/about/guidelines.xhtml

Discussion

The Evaluation of the pre-hospital use of a Valsalva Assist Device in the Emergency treatment of supraventricular tachycardia [EVADE SVT] trial is the first study to assess whether the routine availability of a simple device (VAD) and its instructions to assist the ambulance service delivery of the Valsalva manoeuvre to treat SVT reduces the proportion of patients with SVT conveyed to hospital. In light of detailed feasibility work and patient advice, the study has been specifically designed to be delivered during routine care by normal clinical staff within the pre-hospital setting.

The proposed study is powered to detect a meaningful difference in conveyance rate which would have important benefits for patients and emergency services. Successfully treated patients will feel better sooner and avoid disruption to their lives and potentially long waits and further treatment in the emergency department. If a reduction in conveyance rate is demonstrated when the VAD is made available, the reduced need for patients to be brought to emergency departments would also deliver potential savings for ambulance services that would cover the cost of these devices within seven months and provide patients with an easy-to-use device to keep for self-treatment. Frontline ambulances would also be available sooner to attend other emergencies and emergency departments would have fewer patients to assess and treat.

An innovative trial design (in this setting and challenging environment for research) has been proposed to deliver the aims of the project, whilst maintaining scientific integrity and at a cost commensurate with the condition. The stepped wedge design will enable the randomised introduction of VAD availability as standard care. This ensures patients receive timely emergency care by normal treating pre-hospital clinicians, without additional research burdens, whilst still giving patients the opportunity to contribute to research should they wish to be included. A robust system to ensure patients' prior wishes regarding use of routine clinical data for research are respected and that all patients can opt out should they not wish to take part, ensures that good clinical practice principles are maintained. Importantly, this approach was driven and supported by the project's public and patient engagement work.

For peer review only - http://bmjopen.bmj.com/site/about/guidelines.xhtml

The use of routine data and delivery of the trial during normal care will contribute to study efficiency and help to ensure that study findings will be representative of real-life practice and improve external validity. Service-wide introduction of the intervention during normal practice, with assessment of the overall device use across the trust, will also give some measure of its acceptability and ease of implementation. This will also be useful to other ambulance trusts when considering the study findings.

The primary outcome of the study was carefully considered. Cardioversion was contemplated as an alternative primary outcome, but our feasibility study and other previous research have demonstrated challenges in providing robust evidence and timing of cardioversion. Conveyance is a clear, measurable outcome which is important to patients, clinicians and health services.

The study design means the trial recruitment rate will also be determined by normal practice. Whilst this will ensure all eligible adult patients undergoing a SWASFT-delivered VM can be included in the trial, recruitment could potentially be affected by other factors affecting ambulance availability and wider pressures on the ambulance service. Previous feasibility work demonstrated a consistent incidence of VM treatment, at various time points, including during the COVID pandemic. These data were used to determine the anticipated duration of the stepped wedge phases and overall trial duration. However, there is no certainty that the previously observed incidence rate will be maintained throughout this definitive trial. Recent, well-documented ambulance delays could affect recruitment rates if, for example, more patients make their own way to hospital.

The planned analysis of health-related quality of life (EQ-5D-5L) data through an optional questionnaire will be dependent on response rates to an invitation included in the PIL. Although it is known that response rates to health questionnaires are notoriously low in emergency settings[19], the main trial design and resources for the study do not allow for an alternative approach to collect these data. Recruitment and EQ-5D response rates will therefore be closely monitored and action considered in conjunction with the trial oversight committee, sponsor and funder, as required.

For peer review only - http://bmjopen.bmj.com/site/about/guidelines.xhtml

Conclusions:

The EVADE SVT trial will seek to determine whether the routine availability of a simple device (VAD) and its instructions to assist the ambulance service delivery of the Valsalva manoeuvre to treat SVT reduces the proportion of patients with SVT that are conveyed to hospital. This trial protocol has been developed to deliver this trial within one large UK ambulance service during routine care. It is designed to detect a meaningful change in conveyance rate that would be of benefit to patients and emergency services and be relevant to other ambulance and emergency services around the world.

(Word count 5719 including tables)

For peer review only

For peer review only - http://bmjopen.bmj.com/site/about/guidelines.xhtml

Acknowledgements

We would like to acknowledge and thank all our patient representatives, for their help in writing and reviewing patient facing-materials and in the design of the study and for their views on the protocol and research plan. We would also like to thank Alan McCleod and Izzy Fitzgerald for their support in the development of the VAD.

Patient and Public Involvement Statement

In line with INVOLVE guidelines,[20] we have engaged patients at every stage of the study's design. Our project plan has been discussed widely with three different patient and public groups including our feasibility study patient group, ambulance service patient representatives and the Exeter 10000 (a volunteer group who review trial proposals) and adapted accordingly. There was wide support for our design which was felt to be appropriate, proportionate and respectful of patient views.

Trial Management Group

Dr Andrew Appelboam (Chief Investigator); Dr Sarah Black (Principal Investigator); Alison Coppola (Lead Research Paramedic); Michala Cox (Lead Research Paramedic); Siobhan Creanor (CTU Director); Phoebe Dawes (CTU Data Manager); Ruth Hawker (Patient Representative and Co-applicant); Dr Shelley Rhodes (Senior CTU Trial Manager), Ria Osborne (Trial Manager); Yunli Ou (Trial Statistician); Nicholas Richards (Critical Care Paramedic); Rebecca Summers (Patient and Public Involvement and Engagement Lead); Obi Ukoumunne (Senior Trial Statistician); Annie Hawton (Health Economist).

Trial Steering Committee

Dr Andrew Appelboam (Chief Investigator); Prof Jonathan Benger (Consultant in Emergency Medicine, University Hospitals Bristol NHS Foundation Trust); Dr Clare Thorn (PPI Representative); Diana Frost (PPI Representative); Jon Green (Paramedic Science Programme Lead), Professor Mona Kanaan (Independent Statistician); Professor Sarah Voss (Professor of Emergency Care).

Author Contributions

AA conceived the idea for the study and is lead author, guarantor and chief investigator. RO is the trial manager. AA, NR, NS were responsible for the initial study

For peer review only - http://bmjopen.bmj.com/site/about/guidelines.xhtml

design which was refined with the help of OU, SB, RO and SC and support of our regional NIHR Research Design Service. OU and BJ provided the statistical plan and RH, AG and BS represented the public and patient views during study development and conduct. TC led the design of data management systems, SH and SC are the CTU manager and director respectively and AH has provided health economic advice and support to the project. MD helped set up the ECG review protocol and has provided cardiology advise and oversight for the project.

All authors contributed to the final study design and protocol development, critically revised successive drafts of the manuscript and approved the final version. The Trial Management Group is responsible for the conduct of the study, overseen by a Trial Steering Committee. Contractual agreements are in place between all trial stakeholders including trial data access and use.

For peer review only

Data Statement

During the trial, data will be held and managed by Ex-CTU and retained after the trial by the sponsor. Data will be managed according to the sponsor's policy and available for researchers upon reasonable request.

Funding statement

This research project is funded by the National Institute for Health Research (NIHR) through its Research for Patient Benefit (RfPB) Programme (Grant Reference Number NIHR202185). Ben Jones, Yunli Ou and Obi Ukoumunne were supported by the National Institute for Health and Care Research (NIHR) Applied Research Collaboration (ARC) South West Peninsula. The views expressed are those of the author(s) and not necessarily those of the NIHR or the Department of Health and Social Care.

Competing interests statement

Andrew Appelboam, on behalf of the Royal Devon University Healthcare NHS Foundation Trust (RDUFT), introduced the concept of the Valsalva Assist Device to Meditech Systems Limited and advised on refinements to the device in light of initial volunteer studies. RDUFT has a royalty agreement on future sales of the device. Any money received can only be used for patient care or further research. None of the research team have received or currently stand to gain personal financial benefit from

For peer review only - http://bmjopen.bmj.com/site/about/guidelines.xhtml

Meditech, are shareholders or have any direct financial interest in the company. Meditech has was not involved in the design of the study and will have no role in the conduct, analysis or interpretation of the study nor in the publication of its findings.

Protocol Version:

This paper is based upon the EVADE SVT Study final protocol version 2.1, dated 18/07/22

***EVADE SVT Study Collaborators:**

Michela Cox, Alison Coppola, Beccy Summers, Emma Cockcroft, Yunli Ou, Phoebe Dawe, Leanne Bessell, Ludwig Holzer, Ross Clarkson, Charlotte Macey-Woolmer, Leigh Simpkins, Sarah-Jane Hill, Andy Waring, Will Kivell, Alice Foxen, Samuel Withams, Jade Barber, Tarin Payne, Thomas Boyling, Kristin Houlgate, Dane O'Reilly, Jamie Sprake, Dylan Evans, Peter Chapman, Daniel Titcombe, Gemma Abbott, Peter Caine, Ben Astley, Vince McKenzie-Plume, Adam Hill, Neil Armstrong, Jim Burn, Ashley Ingram, Paula Long, Joe Phipps, Lewis Hare, Kirsty Walker, Charlotte Copley, James Keat, Craig Chedzoy, Leah Mason, David Willis, James King, Victoria Jones, Rich Godfrey, Martyn Morris, Dale Jefferies, Nicole Morrison, Kirsty Carpenter, Steve Palmer, Tara Marchant, Mark Reilly, Sabrina Bolwell, Matthew Adams, Helen Harris, Tracy Hoad, Stuart Stimson, Collette Young, Oscar Miller, Joe Meering, Ashley Watson, William Price, Katharine Laurenson, Lee Clarke, John Carrielies, Sharon Bowyer, Adam Blinman, Anna McCann, Carrie-Anne Durrant, Charlotte Blow, Chris Taplin, Marcus Goodwin, Damon Godfrey, Maree Holmes, Rhys Allsopp, Peter Willows, Katrina Scott, Sonia Wright-Stainton, Stacey Lawrence, Jacquiline Mullarney, Lewis Garrood, Andy Cole, Tom Rutherford, Luke Slocombe, Alan Piper, Julia Ryder, Tom Driver, Mike Smith, Hannah Barber, Joe Cartwright, Loxley Patterson, Chris Birch, Kelly Chappell, James Constable, Naomi Miller, Tara Rodda, Tim Taylor, Jason Trudgill, Russell Hoskin, Harry Brown, Julie Legg, Toni Howell, Fergus Beeley, Zoe Flowerdew, Lucy Davies, Gary Rooney, Cassandra Day, Tamara Hoare, Dylan Carey, Lewis Murphy.

References:

1.Orejarena LA, Vidaillet H, DeStefano F, et al. Paroxysmal supraventricular tachycardia in the general population. J Am Coll Cardiol. 1998;31(1):150-157

2.Wood KA, Drew BJ, Scheinman MM. Frequency of disabling symptoms in supraventricular tachycardia. Am J Cardiol. 1997;79(2):145-9.

3. Smith G, McD Taylor D, Morgans A, Cameron P. Measuring the effectiveness of a revised clinical practice guideline for the pre-hospital management of

For peer review only - http://bmjopen.bmj.com/site/about/guidelines.xhtml

supraventricular tachycardia. Emerg Med Australas. 2015 Feb;27(1):22-8. doi:10.1111/1742-6723.123404.

4. JRCALC Guidelines: https://aace.org.uk/clinical-practice-guidelines/

5. Resuscitation Council (UK) Resuscitation Guidelines 2005. http://www.resus.org.uk/pages/guide.htm

6. Smith G, Morgans, Boyle M. Use of the valsalva manoeuvre in the prehospital setting: a review of the literature. Emerg Med J. 2009;26:8-10

7. Honarbakhsh S, Baker V, Kirkby C, et al Safety and efficacy of paramedic treatment of regular supraventricular tachycardia: a randomised controlled trial Heart 2017;103:1413-1418.

8. Appelboam A, Green J, Ewings P, et al. Evaluation of Pre-hospital use of a Valsalva Assist Device in the Emergency treatment of supraventricular tachycardia [EVADE]: A randomised controlled feasibility trial. Pilot Feasibility Stud 6, 74 (2020). https://doi.org/10.1186/s40814-020-00616-y

9. Smith G, Boyle M. The 10ml syringe is useful in generating the recommended standard of 40mmHg intrathoracic pressure for the Valsalva manoeuvre. Emerg Med Aust 2009;21:449-454

10. European Cardiology Society SVT Guidelines https://www.escardio.org/Guidelines/Clinical-Practice-Guidelines/Supraventricular-Tachycardia Last accessed 27/11/22

11. Appelboam A, Reuben A, Mann C, et al; REVERT Trial Collaborators. Postural modification to the standard Valsalva manoeuvre for emergency treatment of supraventricular tachycardias (REVERT): a randomised controlled trial. Lancet 2015; 31;386:1747–53.

12. Thornton HS, Elwan MH, Reynolds JA, et al Valsalva using a syringe: pressure and variation Emerg Med J 2016;33:748-749.

For peer review only - http://bmjopen.bmj.com/site/about/guidelines.xhtml

13. FitzGerald I, Ewings P, Lang I, Appelboam A. Testing of a novel Valsalva Assist Device with supine and modified positions in healthy volunteers. Emerg Med J. 2019 Jan;36(1):27-31. doi: 10.1136/emermed-2018- 208004. Epub 2018 Dec 1

14. O'Cathain A, Knowles E, Bishop-Edwards L, et al. Understanding variation in ambulance service non-conveyance rates: a mixed methods study. Southampton (UK): NIHR Journals Library; 2018 May. (Health Services and Delivery Research, No. 6.19.) Available from: https://www.ncbi.nlm.nih.gov/books/NBK506846/doi: 10.3310/hsdr06190

15. Chan A-W, Tetzlaff JM, Gøtzsche PC, Altman DG, Mann H, Berlin J, Dickersin K, Hróbjartsson A, Schulz KF, Parulekar WR, Krleža-Jerić K, Laupacis A, Moher D. SPIRIT 2013 Explanation and Elaboration: Guidance for protocols of clinical trials. BMJ. 2013;346:e7586

16. NIHR. NIHR CRN Recruitment Policy Document Version 1.7 2018 [Available from: https://www.nihr.ac.uk/documents/nihr-crn-recruitment-policy-document/11347#21._Assumed_consent accessed 11/10/2021

17. Hussey MA, Hughes JP: Design and analysis of stepped-wedge cluster randomized trials. Contemp Clin Trials 2007, 28:182– 21. 91 Involve Guidelines https://www.invo.org.uk/ Last accessed 15/05/20

18. https://www.swast.nhs.uk/welcome/research-in-south-western-ambulance-service/evade/ Last accessed 03/01/23

19. Mathews M, Parast L, Tolpadi A, et al. "Methods for Improving Response Rates in an Emergency Department Setting – A Randomized Feasibility Study." Survey Practice 12 (1). https://doi.org/10.29115/SP-2019-0007.

20. Involve Guidelines https://www.invo.org.uk/ Last accessed 15/05/20

For peer review only - http://bmjopen.bmj.com/site/about/guidelines.xhtml

Figure 1. The ‘Valsa-Valve’ Valsalva Assist Device packaging and device details

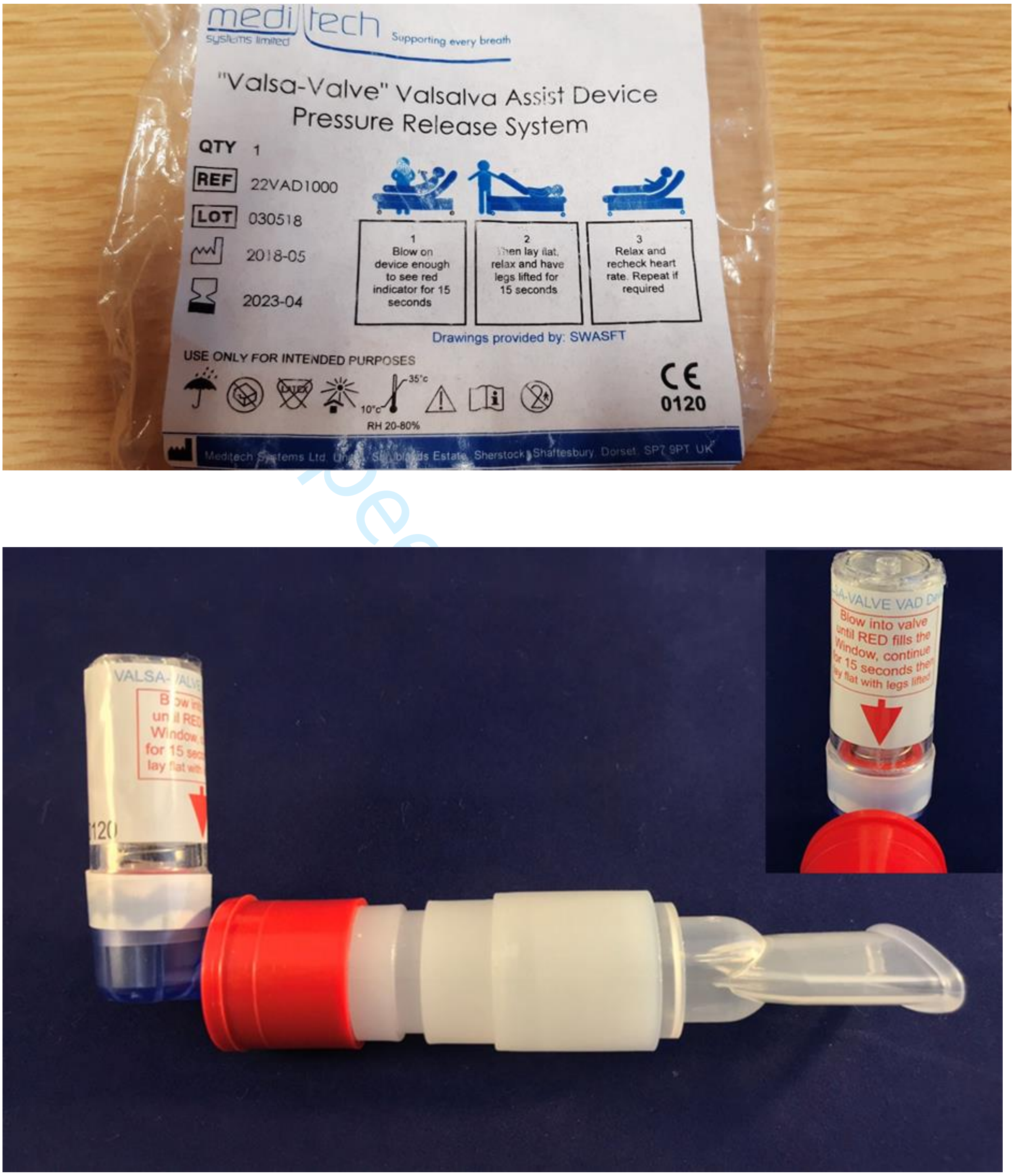

Figure 2. Scheme for the Randomised Stepped Wedge Introduction of the VAD to the Ambulance Service

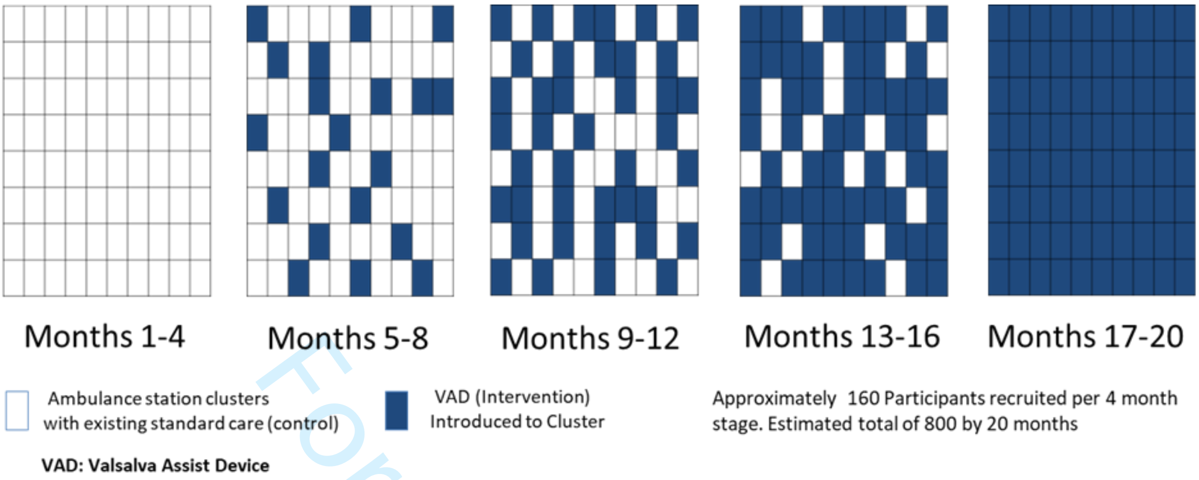

For peer review only

Reporting checklist for protocol of a clinical trial.

Based on the SPIRIT guidelines.

Instructions to authors

Complete this checklist by entering the page numbers from your manuscript where readers will find each of the items listed below.

Your article may not currently address all the items on the checklist. Please modify your text to include the missing information. If you are certain that an item does not apply, please write "n/a" and provide a short explanation.

Upload your completed checklist as an extra file when you submit to a journal.

In your methods section, say that you used the SPIRITreporting guidelines, and cite them as:

Chan A-W, Tetzlaff JM, Gøtzsche PC, Altman DG, Mann H, Berlin J, Dickersin K, Hróbjartsson A, Schulz KF, Parulekar WR, Krleža-Jerić K, Laupacis A, Moher D. SPIRIT 2013 Explanation and Elaboration: Guidance for protocols of clinical trials. BMJ. 2013;346:e7586

		Reporting Item	Page Number
**Administrative information**			
Title	#1	Descriptive title identifying the study design, population, interventions, and, if applicable, trial acronym	1

For peer review only

Trial registration	#2a	Trial identifier and registry name. If not yet registered, name of intended registry	2
Trial registration: data set	#2b	All items from the World Health Organization Trial Registration Data Set	2
Protocol version	#3	Date and version identifier	21
Funding	#4	Sources and types of financial, material, and other support	21
Roles and responsibilities: contributorship	#5a	Names, affiliations, and roles of protocol contributors	1 & 20
Roles and responsibilities: sponsor contact information	#5b	Name and contact information for the trial sponsor	1
Roles and responsibilities: sponsor and funder	#5c	Role of study sponsor and funders, if any, in study design; collection, management, analysis, and interpretation of data; writing of the report; and the decision to submit the report for publication, including whether they will have ultimate authority over any of these activities	21
Roles and responsibilities: committees	#5d	Composition, roles, and responsibilities of the coordinating centre, steering committee, endpoint adjudication committee, data management team, and	20

For peer review only

		other individuals or groups overseeing the trial, if applicable (see Item 21a for data monitoring committee)	
**Introduction**			
Background and rationale	#6a	Description of research question and justification for undertaking the trial, including summary of relevant studies (published and unpublished) examining benefits and harms for each intervention	4-5
Background and rationale: choice of comparators	#6b	Explanation for choice of comparators	4-5
Objectives	#7	Specific objectives or hypotheses	5
Trial design	#8	Description of trial design including type of trial (eg, parallel group, crossover, factorial, single group), allocation ratio, and framework (eg, superiority, equivalence, non-inferiority, exploratory)	5-16
**Methods: Participants, interventions, and outcomes**			
Study setting	#9	Description of study settings (eg, community clinic, academic hospital) and list of countries where data	5

For peer review only

		will be collected. Reference to where list of study sites can be obtained	
Eligibility criteria	#10	Inclusion and exclusion criteria for participants. If applicable, eligibility criteria for study centres and individuals who will perform the interventions (eg, surgeons, psychotherapists)	6
Interventions: description	#11a	Interventions for each group with sufficient detail to allow replication, including how and when they will be administered	5
Interventions: modifications	#11b	Criteria for discontinuing or modifying allocated interventions for a given trial participant (eg, drug dose change in response to harms, participant request, or improving / worsening disease)	7
Interventions: adherance	#11c	Strategies to improve adherence to intervention protocols, and any procedures for monitoring adherence (eg, drug tablet return; laboratory tests)	14
Interventions: concomitant care	#11d	Relevant concomitant care and interventions that are permitted or prohibited during the trial	7
Outcomes	#12	Primary, secondary, and other outcomes, including the specific measurement variable (eg, systolic blood pressure), analysis metric (eg, change from baseline, final value, time to event), method of aggregation (eg, median, proportion), and time point for each outcome.	13

For peer review only

		Explanation of the clinical relevance of chosen efficacy and harm outcomes is strongly recommended	
Participant timeline	#13	Time schedule of enrolment, interventions (including any run-ins and washouts), assessments, and visits for participants. A schematic diagram is highly recommended (see Figure)	8 & 12
Sample size	#14	Estimated number of participants needed to achieve study objectives and how it was determined, including clinical and statistical assumptions supporting any sample size calculations	13-14
Recruitment	#15	Strategies for achieving adequate participant enrolment to reach target sample size	14
**Methods: Assignment of interventions (for controlled trials)**			
Allocation: sequence generation	#16a	Method of generating the allocation sequence (eg, computer-generated random numbers), and list of any factors for stratification. To reduce predictability of a random sequence, details of any planned restriction (eg, blocking) should be provided in a separate document that is unavailable to those who enrol participants or assign interventions	9-11

For peer review only

Allocation concealment mechanism	#16b	Mechanism of implementing the allocation sequence (eg, central telephone; sequentially numbered, opaque, sealed envelopes), describing any steps to conceal the sequence until interventions are assigned	9-11
Allocation: implementation	#16c	Who will generate the allocation sequence, who will enrol participants, and who will assign participants to interventions	10
Blinding (masking)	#17a	Who will be blinded after assignment to interventions (eg, trial participants, care providers, outcome assessors, data analysts), and how	10
Blinding (masking): emergency unblinding	#17b	If blinded, circumstances under which unblinding is permissible, and procedure for revealing a participant's allocated intervention during the trial	10
**Methods: Data collection, management, and analysis**			
Data collection plan	#18a	Plans for assessment and collection of outcome, baseline, and other trial data, including any related processes to promote data quality (eg, duplicate measurements, training of assessors) and a description of study instruments (eg, questionnaires, laboratory tests) along with their reliability and validity,	15-16

For peer review only

		if known. Reference to where data collection forms can be found, if not in the protocol	
Data collection plan: retention	#18b	Plans to promote participant retention and complete follow-up, including list of any outcome data to be collected for participants who discontinue or deviate from intervention protocols	16
Data management	#19	Plans for data entry, coding, security, and storage, including any related processes to promote data quality (eg, double data entry; range checks for data values). Reference to where details of data management procedures can be found, if not in the protocol	16
Statistics: outcomes	#20a	Statistical methods for analysing primary and secondary outcomes. Reference to where other details of the statistical analysis plan can be found, if not in the protocol	15-16
Statistics: additional analyses	#20b	Methods for any additional analyses (eg, subgroup and adjusted analyses)	15-16
Statistics: analysis population and missing data	#20c	Definition of analysis population relating to protocol non-adherence (eg, as randomised analysis), and any statistical methods to handle missing data (eg, multiple imputation)	16

Methods: Monitoring

For peer review only

Data monitoring: formal committee	#21a	Composition of data monitoring committee (DMC); summary of its role and reporting structure; statement of whether it is independent from the sponsor and competing interests; and reference to where further details about its charter can be found, if not in the protocol. Alternatively, an explanation of why a DMC is not needed	16
Data monitoring: interim analysis	#21b	Description of any interim analyses and stopping guidelines, including who will have access to these interim results and make the final decision to terminate the trial	12-13, 14-15
Harms	#22	Plans for collecting, assessing, reporting, and managing solicited and spontaneously reported adverse events and other unintended effects of trial interventions or trial conduct	12-13
Auditing	#23	Frequency and procedures for auditing trial conduct, if any, and whether the process will be independent from investigators and the sponsor	10
**Ethics and dissemination**			
Research ethics approval	#24	Plans for seeking research ethics committee / institutional review board (REC / IRB) approval	16

For peer review only

Protocol amendments	#25	Plans for communicating important protocol modifications (eg, changes to eligibility criteria, outcomes, analyses) to relevant parties (eg, investigators, REC / IRBs, trial participants, trial registries, journals, regulators)	16
Consent or assent	#26a	Who will obtain informed consent or assent from potential trial participants or authorised surrogates, and how (see Item 32)	7-9
Consent or assent: ancillary studies	#26b	Additional consent provisions for collection and use of participant data and biological specimens in ancillary studies, if applicable	8
Confidentiality	#27	How personal information about potential and enrolled participants will be collected, shared, and maintained in order to protect confidentiality before, during, and after the trial	16
Declaration of interests	#28	Financial and other competing interests for principal investigators for the overall trial and each study site	21
Data access	#29	Statement of who will have access to the final trial dataset, and disclosure of contractual agreements that limit such access for investigators	1 & 21
Ancillary and post trial care	#30	Provisions, if any, for ancillary and post-trial care, and for compensation to those who suffer harm from trial participation	12 and covered by

For peer review only

			NHS indemnity
Dissemination policy: trial results	#31a	Plans for investigators and sponsor to communicate trial results to participants, healthcare professionals, the public, and other relevant groups (eg, via publication, reporting in results databases, or other data sharing arrangements), including any publication restrictions	16
Dissemination policy: authorship	#31b	Authorship eligibility guidelines and any intended use of professional writers	16
Dissemination policy: reproducible research	#31c	Plans, if any, for granting public access to the full protocol, participant-level dataset, and statistical code	21
**Appendices**			
Informed consent materials	#32	Model consent form and other related documentation given to participants and authorised surrogates	N/A
Biological specimens	#33	Plans for collection, laboratory evaluation, and storage of biological specimens for genetic or molecular analysis in the current trial and for future use in ancillary studies, if applicable	N/A

None The SPIRIT Explanation and Elaboration paper is distributed under the terms of the Creative Commons Attribution License CC-BY-NC. This checklist can be completed online using https://www.goodreports.org/, a tool made by the EQUATOR Network in collaboration with Penelope.ai

For peer review only

BMJ Open

Evaluation of the pre-hospital use of a Valsalva assist device in the emergency treatment of supraventricular tachycardia (EVADE SVT): study protocol for a stepped wedge cluster randomised controlled trial

Journal:	*BMJ Open*
Manuscript ID	bmjopen-2023-073315.R1
Article Type:	Protocol
Date Submitted by the Author:	05-Apr-2023
Complete List of Authors:	Appelboam, Andrew; Royal Devon University Healthcare NHS Foundation Trust, Emergency Department; University of Exeter<br>Osborne, Ria; South Western Ambulance Service NHS Foundation Trust<br>Ukoumunne, Obioha; University of Exeter, NIHR CLAHRC South West Peninsula (PenCLAHRC)<br>Black, Sarah; South Western Ambulance Service NHS Foundation Trust, Research, Audit and Improvement<br>Boot, Suzanne; South Western Ambulance Service NHS Foundation Trust<br>Richards, Nicolas; South Western Ambulance Service NHS Foundation Trust<br>Scotney, Natalie; South Western Ambulance Service NHS Foundation Trust<br>Rhodes, Shelley; University of Exeter, Medical School<br>Cranston, Tim; University of Exeter, Medical School<br>Hawker, Ruth; South Western Ambulance Service NHS Foundation Trust, PPI Representative, honorary SWASFT contract.<br>Gillett, Annette; University of Exeter Medical School, Peninsula Childhood Disability Research Unit (PenCRU)<br>Jones, Ben; University of Exeter, Medical statistics<br>Hawton, Annie; Peninsula Medical School<br>Dayer, Mark; Taunton and Somerset NHS Foundation Trust, Cardiology<br>Creanor, Siobhan; University of Exeter<br>EVADE SVT study collaborators, EVADE SVT study collaborators; South Western Ambulance Service NHS Foundation Trust
**Primary Subject Heading**:	Emergency medicine
Secondary Subject Heading:	Emergency medicine, Cardiovascular medicine
Keywords:	ACCIDENT & EMERGENCY MEDICINE, Pacing & electrophysiology < CARDIOLOGY, Adult cardiology < CARDIOLOGY

Evaluation of the pre-hospital use of a Valsalva assist device in the emergency treatment of supraventricular tachycardia (EVADE SVT): study protocol for a stepped wedge cluster randomised controlled trial

Andrew Appelboam[1], Ria Osborne[2], Obioha C Ukoumunne[3], Sarah Black[2], Suzanne Boot[2], Nicolas Richards[2], Natalie Scotney[2], Shelley Rhodes[4], Tim Cranston[4], Ruth Hawker[5], Annette Gillet[3], Ben Jones[3], Annie Hawton[6], Mark Dayer[7], Siobhan Creanor[4] and the EVADE SVT study collaborators*

[1]Academic Department of Emergency Medicine, Royal Devon University Healthcare NHS Foundation Trust, Exeter and University of Exeter; [2]South Western Ambulance Service NHS Foundation Trust; [3]NIHR Applied Research Collaboration South West Peninsula, Department of Health and Community Sciences, Faculty of Health and Life Sciences, University of Exeter; [4]Exeter Clinical Trials Unit, University of Exeter; [5]Patient and public representative; [6]Health Economics Group, University of Exeter, [7]Department of Cardiology, Somerset NHS Foundation Trust.; University of Plymouth, Faculty of Health

Corresponding Author: Andrew Appelboam.

c/o Emergency Department, Royal Devon University Healthcare NHS Foundation Trust, Barrack Road, Exeter, Devon EX2 3DW

Tel : 00 44 (0)1392 402170

Fax : 00 44 (0)1392 402313

Email: Andy.appelboam@nhs.net

Trial Sponsor : South Western Ambulance Service NHS Foundation Trust, Abbey Court, Eagle Way, Exeter, EX2 7HY

Keywords : Supraventricular tachycardia, Valsalva manouvre, emergency care

For peer review only - http://bmjopen.bmj.com/site/about/guidelines.xhtml

Abstract

Introduction: Patients with episodes of supraventricular tachycardia (SVT), a common heart arrhythmia, are often attended by ambulance services. International guidelines advocate treatment with the Valsalva Manoeuvre (VM), but this simple physical treatment has a low success rate, with most patients requiring conveyance to hospital. The Valsalva Assist Device (VAD) is a simple device that might help practitioners and patients perform a more effective VM and reduce the need for patients to be taken to hospital.

Methods and analysis: This stepped wedge cluster randomised controlled trial, conducted within a UK ambulance service, compares the current standard VM with a VAD-delivered VM in stable adult patients presenting to the ambulance service with SVT. The primary outcome is conveyance to hospital; secondary outcomes measures include cardioversion rates, duration of ambulance care and number of subsequent episodes of SVT requiring ambulance service care. We plan to recruit approximately 800 patients, to have 90% power to detect an absolute reduction in conveyance rate of 10% (from 90% to 80%) between the standard VM (control) and VAD-delivered VM (intervention). Such a reduction in conveyance would benefit patients, the ambulance service and receiving emergency departments. It is estimated potential savings would pay for devices for the entire ambulance trust within seven months.

Ethics and dissemination: The study has been approved by the Oxford Research Ethics Committee (reference 22/SC/0032). Dissemination will be through peer-reviewed journal publication, presentation at national and international conferences and by the Arrhythmia Alliance, a patient support charity.

Registration: Current Controlled Trials (ISRCTN 16145266). Adopted by the National Institute for Health Research (NIHR) Clinical Research Network.

Abstract word count: 264

For peer review only

For peer review only - http://bmjopen.bmj.com/site/about/guidelines.xhtml

Article Summary

Strengths and limitations of this study

- The efficient, pragmatic design of the trial and use of routine data, enables delivery of the study during normal ambulance service care of patients with SVT, as the device is brought into standard use.
- The study is designed to detect a difference in conveyance rate; a reliable outcome that is of importance to patients, the ambulance service and receiving hospitals.
- Although individual participant randomisation and blinding is not possible in this study, blinding of the trial statisticians will be maintained until the statistical analysis plan is finalised and signed off by the Trial Steering Committee.

For peer review only

For peer review only - http://bmjopen.bmj.com/site/about/guidelines.xhtml

Introduction

Supraventricular tachycardias (SVT) are a common heart rhythm disorder causing episodes of fast heart rates.. The incidence of SVT is estimated to be 35 episodes per 100,000 persons/year [1] and attacks are unpleasant and disruptive to patients' lives [2]. SVT often occurs because of an additional electrical pathway in the heart, causing a 're-entrant tachycardia'.

Pre-hospital emergency services often attend patients with attacks of SVT. A regional Australian ambulance service recorded seeing 800 cases of SVT in a year [3] with audit data suggesting a similar number of episodes in the South Western Ambulance Service NHS Foundation Trust (SWASFT), in the South West of England, UK. International ambulance treatment guidelines recommend initial treatment with the Valsalva Manoeuvre (VM) [4],[5]. This simple physical treatment involves an exhalation strain against resistance which causes a slowing of the heart (mediated by the vagal nerve) and can cardiovert an SVT back to normal sinus rhythm. Unfortunately, cardioversion rates in routine pre-hospital practice can be as low as 5% and most patients are conveyed to hospital with ongoing SVT [6],[7].

Results from our feasibility study suggested SWASFT ambulance clinicians treat around 500 of the SVT episodes they see each year, using a VM with approximately 90% of these patients being conveyed to hospital [8]. This ambulance service has previously adopted the modified Valsalva manoeuvre (mVM) into its guidelines as the first-line treatment in stable patients with SVT. This guideline, in common with others, recommends that the VM strain is delivered by blowing into a 10 ml syringe [9] followed by an internationally recommended [10] postural modification which has shown to improve cardioversion rates [11]. However, syringes are unreliable in generating the correct VM strain pressure [12] and remembering the modified manoeuvre may be difficult for individual clinicians, who only occasionally see these patients.

A simple, easy to use Valsalva Assist Device (VAD) has been developed which provides the correct, recommended resistance (40mmHg) for the VM exhalation strain [13]. A CE-marked VAD has been produced by Meditech Systems Limited (www.meditechsystems.co.uk), marketed as the 'Valsa-Valve' Valsalva Assist Device (see figure 1). This VAD includes instructions for use on the device and it's packaging, including information on the timings and the postural changes required for the mVM.

For peer review only - http://bmjopen.bmj.com/site/about/guidelines.xhtml

If appropriate, these single-patient-use devices can also be left with the patient, for subsequent use should an attack recur. The VAD might improve the quality of the VM and increase the chances of cardioversion and non-conveyance compared to the standard approach using syringe delivered VMs. Reducing ambulance conveyance of patients to hospital, when appropriate and possible, is increasingly important to health services and patients [14].

A definitive randomised controlled trial is needed to test whether the availability of a VAD to ambulance clinicians, when treating patients with SVT, affects the success of treatment and the need for conveyance to hospital, compared to current practice. A recent feasibility study and patient and public feedback have informed the nature and design of the definitive trial outlined in this protocol paper.

Methods and analysis

General study design and conduct:

This study is an efficient, pragmatic, stepped wedge cluster randomised controlled trial to see if the routine availability of a VAD and its instructions (intervention), to treat adult patients presenting to SWASFT ambulance clinicians with SVT, can reduce the rate of hospital conveyance compared with standard practice (control). The proposed stepped wedge design involves the allocation of ambulance service clusters across the whole service. These clusters all start as controls, providing current standard care, before being randomly assigned to using the VAD, as a new standard treatment, in defined groups and periods over the duration of the study (further details provided below). This study protocol is reported according to SPIRIT reporting guidelines [15] .

Study population and setting:

Eligible patients presenting to SWASFT with suspected SVT and who are treated with a VM by a SWASFT ambulance clinician, will be included. The study will be conducted entirely within SWASFT, UK. This ambulance service uses a searchable electronic patient care record (ePCR) as standard when attending patients. This was shown to be a reliable and easily searchable data source in our feasibility study. SWASFT also has an established clinical guideline advocating the use of a modified VM and non-conveyance of successfully treated SVT patients.

For peer review only - http://bmjopen.bmj.com/site/about/guidelines.xhtml

The study aims to include all adult patients aged 18 years and over for whom there is a record of a Valsalva Manoeuvre delivered by an ambulance clinician, working out of a participating ambulance station, for presumed SVT.

Patients who subsequently withdraw consent for use of their data or have an existing general opt-out for use of routine NHS data for research will be excluded from the trial. Data from patients who are prisoners at the time of treatment, patients with permanent lack of capacity as recorded by the treating clinician and patients solely treated by a SWASFT member of staff outside of the included ambulance station clusters (e.g., Air Ambulance, Hazardous Area Response Team, British Association for Immediate Care (BASICS) Doctor) will also be excluded. Data from patients previously included in the study will be excluded from the primary outcome analysis but will be used in analyses of some secondary outcomes. A summary table of the trial eligibility criteria is shown in Table 1.

Table 1: Trial Inclusion and Exclusion Criteria

Inclusion criteria	• Adult patients (age 18 years or over)<br>• Record of a Valsalva manoeuvre for presumed SVT having been delivered by an ambulance clinician asigned to a participating ambulance station cluster
Exclusion criteria	• Subsequent withdrawal of patient consent to use their data<br>• Existing opt-out for use of NHS data for research<br>• Data from patients who are prisoners at the time of treatment<br>• Patients with permanent lack of capacity as recorded by the treating clinician<br>• Patients solely treated by a SWASFT resource not from one of the included ambulance station clusters e.g. Air Ambulance, Hazardous Area Response Team,Tiverton Minor Injuries Unit, British Association for Immediate Care (BASICS) Doctor<br>• Data from patients previously included in the study will be excluded from the primary outcome analysis but will be used in analyses of some secondary outcomes

Study procedures:

For peer review only - http://bmjopen.bmj.com/site/about/guidelines.xhtml

Potential study participants will undergo standard clinical assessment by ambulance clinicians. For suspected SVT this typically includes medical history, recording of routine initial observations of pulse, blood pressure, respiratory rate, oxygen saturation and the recording of a 12-lead electrocardiogram (ECG). Routinely collected clinical data are entered into the ePCR system and so are available for subsequent trial data extraction for eligible participants.

The screening of eligibility for VM treatment, the conduct of the VM and all other aspects of clinical care will be entirely governed by SWASFT clinical guidelines, interpreted and followed at the discretion of the treating clinician. On commencement of the intervention phase of the trial, SWASFT clinical guidelines will be updated to include the use of the VAD, where available as part of standard care.

Trial treatment:

The stocking of ambulances with the VAD (and thus the allocation of interventions) will be determined by the randomisation schedule as described below. This will ensure that a VAD is available for use by the treating clinician according to the randomisation schedule. The use of devices will thereafter be at the discretion of the treating clinician but encouraged through trial training and publicity; device use will be recorded and used as a measure of device acceptability.

Post-treatment assessment usually includes the recording of a further 12-lead ECG to determine whether normal sinus rhythm has been restored. However, this is not universal and often omitted if there has been no clear change in the heart rate and rhythm. SWASFT guidelines allow for non-conveyance in stable, well patients who have cardioverted, have normal observations and remain in sinus rhythm after 20 minutes of observation. In this case, patients are advised to seek further follow-up with their general practitioner. Patients will also be given the device (syringe or VAD) they used to perform the VM to take away, with written instructions, in case of recurrent SVT attacks.

Consent:

All participants will be given a participant information leaflet (PIL), specific to the stage of the trial, at a convenient time after treatment, at the discretion of the treating ambulance clinician. The front of the PIL will clearly state that the information can be

For peer review only - http://bmjopen.bmj.com/site/about/guidelines.xhtml

read when the patient is ready and feels well enough. The PIL contains an explanation of SVT, the nature of the trial and how to opt out, should they not want their data to be used. Ambulance clinicians will be asked to record in the ePCR, confirmation that the PIL was given to the patient or a carer. As part of the screening and data entry process, the Lead Research Paramedic will confirm whether a PIL was given by checking the ePCR and, if not recorded, will contact the treating paramedic to confirm. If there is no evidence that a PIL was given at the time of treatment, the Lead Research Paramedic will post a PIL to the patient.

For patients with temporary lack of capacity (for example due to their condition or associated illness) the PIL will be given to the next of kin or carer, if available, to explain and share with the patient when they are well enough. Patients with permanent severe lack of capacity, as determined by the treating ambulance clinician, will not be included. PIL translations will be sent to non-English speakers by SWASFT research staff where possible.

The PIL gives details of how patients can opt out of their routine data being used in the trial. This can be through immediate verbal opt-out (to the treating ambulance clinician) or retrospectively by telephone, post or email. Immediate verbal opt-outs will be recorded on the ePCR and screening of cases for inclusion in the trial dataset will include a full search for opt-outs in the ePCR or received by any other means. An opt-out received at any time during the trial will render the patient ineligible, and their clinical data for their initial and subsequent episodes will not be included in the study. Safety follow-up data, already recorded up to the point of withdrawal, will be kept.

In the UK, patients also have the option to nationally opt out of having any of their routine digital NHS data used for research. The national rate of this type of opt-out currently stands at around 5%. After taking advice from the regional research ethics committee and our public and patient advisory group, we have elected to exclude all patients with a national data opt out unless the patient advises that they want to be included in the study (details of how to do this are included in the PIL).

In the absence of (i) the patient having a national opt-out and (ii) the research team receiving any individual opt-out, inferred consent for routine data use will be assumed. There is a precedent for this approach to collecting routine data for emergency

For peer review only - http://bmjopen.bmj.com/site/about/guidelines.xhtml

medicine research and it is considered to constitute active recruitment as stated in the NIHR Clinical Research Network (CRN) recruitment policy document [16].

Individual trial participation will last from the start of the SVT ambulance service care episode until three months after this time to enable the collection of further ambulance service use data as part of the planned recurrent SVT and safety follow-up period.

Health-related quality of life data:

The PIL also includes an invitation to an optional questionnaire to collect (non-routine) data on the effects of the participant's condition and ambulance service treatment on their health-related quality of life. The invitation includes a web link (with a QR code) to this part of the study, printed on the PIL. Those who choose to participate will be given further information and asked for consent if they follow this link. Once they have given consent, they will be asked to complete the EQ-5D-5L questionnaire online, ideally within a week of the patient feeling well enough to complete the form; this will also be explained in the PIL. Participants who consent to this study component will be contacted digitally and re-invited to complete the EQ-5D-5L, three months after the SVT ambulance service care episode. Data from completed forms will be linked with the study database entry for the index presentation. Paper or emailed copies of the EQ-5D-5L will be sent to participants if requested, as outlined in the PIL. Paper or online EQ-5D-5L will be used under licence.

Formation of ambulance station clusters and randomisation:

Ambulance station clusters will be formed from the 93 SWASFT ambulance stations in existence at the time of study setup. Recent historic data on activity and general conveyance rates for all stations were collected to inform the formation of trial clusters and sample size calculations. After merging very small and closely located stations, whilst maintaining the largest possible number of similar sized clusters, 80 (mostly single station) ambulance station clusters were identified. Clinicians working from these stations use equipment bags strictly belonging to and stocked by that station.

The stepped wedge trial design is comprised of five stages, with each stage lasting four months. In the first stage, none of the station clusters will have access to the VAD and patients will be treated as per standard practice. The clusters will then be randomly allocated, by computer-generated sequences (stratified by location and balanced on

For peer review only - http://bmjopen.bmj.com/site/about/guidelines.xhtml

cluster size), to include VADs with their equipment bags, in one of the 4 remaining stages, so that 20 clusters and their clinicians receive access to the VAD for the first time at each stage. Randomisation will be performed by an independent statistician otherwise unconnected with the trial.

Once the VAD has been introduced, the cluster will retain the use of devices for the remainder of the study so that by the fifth stage, all 80 clusters and their clinicians will have access to the VAD (see Figure 2). Devices will be added to a clearly marked 'Valsalva pouch' attached to the standard equipment bag. These bags are checked and re-stocked daily and carried by all treating ambulances. All station clusters randomised to VAD use will initiate availability of the VAD on the same day, coordinated by SWASFT supply logistics and the local ambulance station 'champions' (listed as trial collaborators). These processes will be overseen by the Clinical Trials Unit (CTU) to ensure protocol compliance as far as possible. Ambulance stations will be notified of activation with sufficient time to enable Valsalva pouches to be changed and all station clinicians to be notified. No other individuals outside the Lead Research Paramedic, logistics supply chain and the station clinicians will be made aware of the randomisation.

The timing and duration of cluster activation will only be changed in response to unexpected (high or low) rates of participant recruitment and only by agreement of the Trial Management Group (TMG), Trial Steering Committee (TSC) and sponsor.

For peer review only

To maintain blinding of research staff, all station clusters will be given a unique code that will used in the clinical report form (CRF). A specific protocol (see below) will also be used to accurately identify the treating cluster (and hence treatment allocation) at the time of patient participation.

EVADE Cluster Identification Protocol:

The process for determining the origin of the attending ambulance (and hence the equipment bag available to the treating clinician and allocation) will be according to a strict protocol. This will ascribe the correct cluster assignment whilst maintaining researcher blinding. Once the cluster is identified, it will only be identified in the CRF by its unique trial ID number. The hierarchy for determining cluster identification (and therefore, the intention-to-treat treatment allocation) is summarised below. Where more than one crew have attended a participant, it will be the first treating crew (as long as they are from a participating station) that will be used to identify the station allocation. The actual treatment used (as recorded in the ePCR) will also be recorded to inform any per protocol analysis.

The treating cluster will be identified by:

1. Determining in the ePCR, which station the standard equipment bag originated from.
2. If the station / cluster cannot be confirmed in this way, the lead clinicians involved will be emailed (likely one of two possible stations).
3. Failing that, station champions will be emailed to request local inspection of drugs books (again likely to be one of two possible stations).
4. Finally, if the cluster cannot be identified through the above steps, support will be requested from the SWASFT Clinical Information and Records Office to access the computer assisted dispatch (CAD) system to provide geographical location coordinates but not address to identify the most likely treating cluster. If required, the Global Rostering System (GRS) may also be used to identify which ambulance stations clinicians were working from.

For peer review only - http://bmjopen.bmj.com/site/about/guidelines.xhtml

Independent ECG review:

To estimate diagnostic appropriateness, a review of available pre-VM ECGs from the ePCR will be undertaken by an independent assessor (consultant electrophysiologist) blind to treatment allocation. Traces with severe interference, preventing interpretation, will be excluded. All other traces will be classified as eligible for VM (e.g., re-entrant SVT, undetermined SVT) or ineligible VM (e.g., atrial fibrillation, atrial flutter, sinus tachycardia). In practice, for normal treating clinicians, it can sometimes be challenging to distinguish between some of these ECG rhythms and VM is appropriate and safe if clinicians believe, on the evidence available, that the patient has an eligible SVT.

Safety monitoring:

The VM is a very safe, standard intervention with no evidence of harm in previous large studies. There is no reason to believe the trial intervention will pose any additional risks or significant delay in the patient's treatment. However, any problems or adverse events during any VM or in the care of trial patients will be recorded, closely monitored and reviewed by the TMG and TSC. Adverse events will be recorded by the pre-hospital clinician in the 'Datix' reporting system used by SWASFT, screened for during data entry and brought to the attention of the Lead Research Paramedic for further consideration. Further ambulance attendances over the following three months from the index attendance will also be reviewed and monitored as part of trial safety monitoring.

An adverse event is classified as a Serious Adverse Event (SAE) if it results in death, is life-threatening, requires hospitalisation or prolongation of existing hospitalisation, results in persistent or significant disability or incapacity, causes a congenital anomaly or is considered by the investigator to be an important medical event. Admission to hospital required for the presenting SVT or associated condition will not be considered an SAE in this trial. However, the reason for admission will be recorded and any admissions that are not clearly related to the participant's presenting condition will be reviewed to assess whether they should be recorded as an SAE.

Any SAE recorded through the Datix system will also be brought to the attention of the Chief Investigator, who will assess it for possible causality linked to the trial in any way.

For peer review only

For peer review only - http://bmjopen.bmj.com/site/about/guidelines.xhtml

All SAEs will be reported to the sponsor and reviewed by the TSC. The trial may be stopped if any indication of harm is found.

Outcome measures:

Primary Outcome: The primary outcome of the study will be the percentage of participants treated with a Valsalva manoeuvre (VM) by the ambulance service, who are conveyed to hospital, as determined by the SWASFT electronic patient care record (ePCR). This includes a mandatory record of the participant's final destination for each care episode. The reasons for conveyance will also be recorded.

Secondary Outcomes: These will include the number of VM attempts used, evidence of postural modification, cardioversion (return to normal heart rhythm) as recorded by the treating ambulance clinician (including spontaneous cardioversion), total time of the ambulance episode and further ambulance attendances for SVT including subsequent conveyance to hospital (to rule out the possibility that treatment postpones attendance to hospital) during the three month period after the index presentation. Other outcome measures will include an estimate of diagnostic appropriateness (through the retrospective expert reading of available, interpretable pre-Valsalva ECG traces recorded in the ePCR) and health-related quality of life questionnaire (EQ-5D-5L) data collected from the optional online link, where available. Compliance with conveyance guidelines will be recorded and also, as part of the safety monitoring for the trial, ambulance attendance for any reason within three months of index presentation will be reported and monitored.

Other measures

Confirmation of the Valsalva strain method used will be recorded. Demographic data, including ethnicity, will also be extracted from the ePCR to describe the extent to which the two groups (intervention versus control) are similar with respect to patient characteristics and that all patient groups, commensurate with the local population demographic, have the opportunity to take part in the research.

Determination of sample size:

The sample size was calculated using formulae presented by Hussey and Hughes [17]. Assuming a baseline non-conveyance rate of 10% (from routine and feasibility study data[8]) and a 10% point increase in non-conveyance with VAD, an intra-cluster

For peer review only - http://bmjopen.bmj.com/site/about/guidelines.xhtml

correlation coefficient of 0.01 and 90% power at the two-sided 5% level of significance, we estimated that the 80 ambulance clusters will need to recruit a total of 800 participants (without repeat presentations) to the trial. The intra-cluster correlation coefficient was estimated to be 0.009 for non-conveyance for all reasons (not just supraventricular tachycardia) using routine data from 94 SWASFT ambulance stations for care episodes for the 12 months between 1st February 2019 and 31st January 2020, inclusive.

Each ambulance cluster would be expected to recruit two participants in each of the five stages (each stage lasts four months) over the 20 months of recruitment. The target effect size was based on the minimum clinically meaningful effect that would justify a change in practice, taking into account the benefits and cost of the devices whilst remaining plausible according to the feasibility trial data (12% absolute increase in non-conveyance (95% confidence interval (CI): 3-34[8])). Prior to commencement of the trial, we also undertook a data collection exercise, using the proposed trial eligibility and data collection methods. This showed 63 (95% CI: 50-77) eligible patients had undergone a VM for SVT in SWASFT over one month. This number was in keeping with data from our feasibility study and previous service evaluations and in excess of that needed in our sample size estimates (40/month). This will allow for repeat presentations (around 10% of all cases by the end of an SVT VM trial of similar duration) and opt-out of data use, which, from our Patient and Public Involvement and Engagement (PPIE) and feasibility work, we expect to be low.

Due to the study design, recruitment will also largely be determined by normal practice rather than specific trial activities. It was felt that 20 months to recruit 800 participants is conservative and achievable. However, initial study progress criteria are proposed to monitor the recruitment rate (see Table 2).

Progress (RAG) Criteria: The stepped wedge design is predicated on reliably recruiting the number of participants specified by the sample size calculation at each stage of the trial. Monthly recruitment will be monitored closely by the TMG and regularly reported to the TSC. Recruitment below target (amber or red criteria) will trigger a remedial plan by the TMG with promotion of the SVT guideline and trial awareness and feedback on missed cases (where a VM was indicated but not undertaken) in addition to the planned promotion of the trial (newsletters, posters, etc.) and NIHR

For peer review only - http://bmjopen.bmj.com/site/about/guidelines.xhtml

CRN support. Financial constraints would preclude significant trial prolongation and if, despite these measures, trial recruitment was less than 80% of our overall target for the first four months of the study, we propose discussion with the TMG, TSC and funder to consider trial termination or agreed modification.

Table 2: Study Progression Criteria

Progression Criteria At 4 Months

Criteria	**Green**	**Amber**	**Red**
Individual-level recruitment	≥ 160 participants (100+%)	128 to 159 participants (80-99%)	< 128 participants (below 80%)
Cluster-level recruitment	80 (100%) clusters each recruit at least one participant	72 to 79 clusters each recruit at least one participant (90-99%)	< 72 clusters each recruit at least one participant (below 90%)

Data analysis:

Analysis will be conducted according to a pre-specified statistical analysis plan (SAP) written by the trial statisticians, approved by an independent statistician on the trial steering committee and made publicly available ahead of database lock.

Participant and cluster (ambulance stations) characteristics will be summarised using means and standard deviations or medians and inter-quartile ranges for continuous variables and numbers and percentages for categorical variables, by condition status (intervention versus control) and overall.

In the main analyses, outcomes will be analysed using the intention-to-treat principle, based on intervention status of the cluster at the time of the VM, irrespective of whether there was evidence of device use. Per protocol analyses will also be undertaken. Repeat presentations of patients previously recruited will be excluded from the primary analyses but included in secondary analyses. The intervention effect will be estimated through fitting mixed effects ("multilevel") regression models that allow for the correlation between outcomes of patients from the same cluster (station). The models will include fixed effects for the intervention effect and time (stage) and a random effect for the cluster.

For peer review only - http://bmjopen.bmj.com/site/about/guidelines.xhtml

Given the nature of the primary outcome and normally well-recorded routine data to be used in the trial, it is not anticipated that there will be a significant quantity of missing data. The trial Research Paramedic will make every attempt to retrieve any missing data. Should any key outcome data remain missing, sensitivity analyses may be proposed, using a range of assumptions where necessary, and consideration given to suitable imputation.

Data monitoring:

All data will be managed according to the trial data management plan. Although no formal data monitoring committee is proposed, overall conveyance rates and adverse event data will be monitored by the TSC to ensure appropriateness and safety. An independent sub-group of the TSC will also assess unblinded conveyance and safety data by group allocation.

Ethics and dissemination

The study was given Regional ethics committee (REC) and Health Research Authority (HRA) approval on 29th April 2022 (22/SC/0032) and complies with the Declaration of Helsinki and GCP guidelines.

The study results will be applicable and of interest to paramedics, ambulance trusts, emergency physicians, acute physicians, general practitioners and patients. We aim to disseminate the study findings to these groups through publication of the trial in appropriate international peer-reviewed journals according to the trial's publication plan. Results will also be presented at appropriate local, national and international academic meetings such as the College of Emergency Medicine scientific meeting and International Conference of Emergency Medicine, as well as the 999 Emergency Medical Services Research Forum and through appropriate press releases and social media.

Patients recruited into the trial will have the opportunity to see the study's findings through the website of the Arrhythmia Alliance, a heart rhythm charity and one of our collaborators, who have offered to post the trial results. Details of this organisation are included in the trial PIL. We will also present the trial on a publicly available study webpage [18] of the SWASFT website.

For peer review only - http://bmjopen.bmj.com/site/about/guidelines.xhtml

Patient and Public Involvement Statement

In line with INVOLVE guidelines [19], we have engaged patients at every stage of the study's design. Our project plan has been discussed widely with three different patient and public groups including our feasibility study patient group, ambulance service patient representatives and the Exeter 10000 (a volunteer group who review trial proposals) and adapted accordingly. There was wide support for our design which was felt to be appropriate, proportionate and respectful of patient views.

Discussion

The Evaluation of the pre-hospital use of a Valsalva Assist Device in the Emergency treatment of supraventricular tachycardia [EVADE SVT] trial is the first study to assess whether the routine availability of a simple device (VAD) and its instructions to assist the ambulance service delivery of the Valsalva manoeuvre to treat SVT reduces the proportion of patients with SVT conveyed to hospital. In light of detailed feasibility work and patient advice, the study has been specifically designed to be delivered during routine care by normal clinical staff within the pre-hospital setting.

The proposed study is powered to detect a meaningful difference in conveyance rate which would have important benefits for patients and emergency services. Successfully treated patients will feel better sooner and avoid disruption to their lives and potentially long waits and further treatment in the emergency department. If a reduction in conveyance rate is demonstrated when the VAD is made available, the reduced need for patients to be brought to emergency departments would also deliver potential savings for ambulance services that would cover the cost of these devices within seven months and provide patients with an easy-to-use device to keep for self-treatment. Frontline ambulances would also be available sooner to attend other emergencies and emergency departments would have fewer patients to assess and treat.

An innovative trial design (in this setting and challenging environment for research) has been proposed to deliver the aims of the project, whilst maintaining scientific integrity and at a cost commensurate with the condition. The stepped wedge design will enable the randomised introduction of VAD availability as standard care. This

For peer review only - http://bmjopen.bmj.com/site/about/guidelines.xhtml

ensures patients receive timely emergency care by normal treating pre-hospital clinicians, without additional research burdens, whilst still giving patients the opportunity to contribute to research should they wish to be included. A robust system to ensure patients' prior wishes regarding use of routine clinical data for research are respected and that all patients can opt out should they not wish to take part, ensures that good clinical practice principles are maintained. Importantly, this approach was driven and supported by the project's public and patient engagement work.

The use of routine data and delivery of the trial during normal care will contribute to study efficiency and help to ensure that study findings will be representative of real-life practice and improve external validity. Service-wide introduction of the intervention during normal practice, with assessment of the overall device use across the trust, will also give some measure of its acceptability and ease of implementation. This will also be useful to other ambulance trusts when considering the study findings.

The primary outcome of the study was carefully considered. Cardioversion was contemplated as an alternative primary outcome, but our feasibility study and other previous research have demonstrated challenges in providing robust evidence and timing of cardioversion. Conveyance is a clear, measurable outcome which is important to patients, clinicians and health services.

The study design means the trial recruitment rate will also be determined by normal practice. Whilst this will ensure all eligible adult patients undergoing a SWASFT-delivered VM can be included in the trial, recruitment could potentially be affected by other factors affecting ambulance availability and wider pressures on the ambulance service. Previous feasibility work demonstrated a consistent incidence of VM treatment, at various time points, including during the COVID pandemic. These data were used to determine the anticipated duration of the stepped wedge phases and overall trial duration. However, there is no certainty that the previously observed incidence rate will be maintained throughout this definitive trial. Recent, well-documented ambulance delays could affect recruitment rates if, for example, more patients make their own way to hospital.

The planned analysis of health-related quality of life (EQ-5D-5L) data through an optional questionnaire will be dependent on response rates to an invitation included in

For peer review only - http://bmjopen.bmj.com/site/about/guidelines.xhtml

the PIL. Although it is known that response rates to health questionnaires are notoriously low in emergency settings [20], the main trial design and resources for the study do not allow for an alternative approach to collect these data. Recruitment and EQ-5D response rates will therefore be closely monitored and action considered in conjunction with the trial oversight committee, sponsor and funder, as required.

Conclusions:

The EVADE SVT trial will seek to determine whether the routine availability of a simple device (VAD) and its instructions to assist the ambulance service delivery of the Valsalva manoeuvre to treat SVT reduces the proportion of patients with SVT that are conveyed to hospital. This trial protocol has been developed to deliver this trial within one large UK ambulance service during routine care. It is designed to detect a meaningful change in conveyance rate that would be of benefit to patients and emergency services and be relevant to other ambulance and emergency services around the world.

(Word count 5719 including tables)

For peer review only

Acknowledgements

We would like to acknowledge and thank all our patient representatives, for their help in writing and reviewing patient facing-materials and in the design of the study and for their views on the protocol and research plan. We would also like to thank Alan McCleod and Izzy Fitzgerald for their support in the development of the VAD.

Trial Management Group

Dr Andrew Appelboam (Chief Investigator); Dr Sarah Black (Principal Investigator); Alison Coppola (Lead Research Paramedic); Michala Cox (Lead Research Paramedic); Siobhan Creanor (CTU Director); Phoebe Dawes (CTU Data Manager); Ruth Hawker (Patient Representative and Co-applicant); Dr Shelley Rhodes (Senior CTU Trial Manager), Ria Osborne (Trial Manager); Yunli Ou (Trial Statistician); Nicholas Richards (Critical Care Paramedic); Rebecca Summers (Patient and Public Involvement and Engagement Lead); Obi Ukoumunne (Senior Trial Statistician); Annie Hawton (Health Economist).

Trial Steering Committee

Dr Andrew Appelboam (Chief Investigator); Prof Jonathan Benger (Consultant in Emergency Medicine, University Hospitals Bristol NHS Foundation Trust); Dr Clare Thorn (PPI Representative); Diana Frost (PPI Representative); Jon Green (Paramedic Science Programme Lead), Professor Mona Kanaan (Independent Statistician); Professor Sarah Voss (Professor of Emergency Care).

Author Contributions

AA conceived the idea for the study and is lead author, guarantor and chief investigator. RO is the trial manager. AA, NR, NS were responsible for the initial study design which was refined with the help of OU, SB, RO and SC and support of our regional NIHR Research Design Service. OU and BJ provided the statistical plan and RH and AG represented the public and patient views during study development and conduct. TC led the design of data management systems, SR and SC are the CTU manager and director respectively and AH has provided health economic advice and support to the project. MD helped set up the ECG review protocol and has provided cardiology advice and oversight for the project. SBo helped with set up of the study

For peer review only - http://bmjopen.bmj.com/site/about/guidelines.xhtml

For peer review only

across the Trust. ESSC advised on station set up and planning of logistical arrangements, acting as station champions to implement the study locally.

All authors contributed to the final study design and protocol development, critically revised successive drafts of the manuscript and approved the final version. The Trial Management Group is responsible for the conduct of the study, overseen by a Trial Steering Committee. Contractual agreements are in place between all trial stakeholders including trial data access and use.

AA – Andrew Appelboam
RO – Ria Osborne
NR – Nick Richards
NS – Natalie Scotney
OU - Obioha Ukoumunne
SB – Sarah Black
SC – Siobhan Creanor
SR – Shelley Rhodes
BJ – Benjamin Jones
RH – Ruth Hawker
AG – Annette Gillet
TC – Tim Cranston
AH – Annie Hawton
MD – Mark Dayer
SBo – Suzanne Boot
ESSC – EVADE SVT study collaborators

For peer review only

Data Statement

During the trial, data will be held and managed by Ex-CTU and retained after the trial by the sponsor. Data will be managed according to the sponsor's policy and available for researchers upon reasonable request.

Funding statement

This research project is funded by the National Institute for Health Research (NIHR) through its Research for Patient Benefit (RfPB) Programme (Grant Reference Number NIHR202185). Ben Jones, Yunli Ou and Obi Ukoumunne were supported by the

For peer review only - http://bmjopen.bmj.com/site/about/guidelines.xhtml

National Institute for Health and Care Research (NIHR) Applied Research Collaboration (ARC) South West Peninsula. The views expressed are those of the author(s) and not necessarily those of the NIHR or the Department of Health and Social Care.

Competing interests statement

Andrew Appelboam, on behalf of the Royal Devon University Healthcare NHS Foundation Trust (RDUFT), introduced the concept of the Valsalva Assist Device to Meditech Systems Limited and advised on refinements to the device in light of initial volunteer studies. RDUFT has a royalty agreement on future sales of the device. Any money received can only be used for patient care or further research. None of the research team have received or currently stand to gain personal financial benefit from Meditech, are shareholders or have any direct financial interest in the company. Meditech has was not involved in the design of the study and will have no role in the conduct, analysis or interpretation of the study nor in the publication of its findings.

Protocol Version:

This paper is based upon the EVADE SVT Study final protocol version 2.1, dated 18/07/22

***EVADE SVT Study Collaborators:**

Michela Cox, Alison Coppola, Beccy Summers, Emma Cockcroft, Yunli Ou, Phoebe Dawe, Leanne Bessell, Ludwig Holzer, Ross Clarkson, Charlotte Macey-Woolmer, Leigh Simpkins, Sarah-Jane Hill, Andy Waring, Will Kivell, Alice Foxen, Samuel Withams, Jade Barber, Tarin Payne, Thomas Boyling, Kristin Houlgate, Dane O'Reilly, Jamie Sprake, Dylan Evans, Peter Chapman, Daniel Titcombe, Gemma Abbott, Peter Caine, Ben Astley, Vince McKenzie-Plume, Adam Hill, Neil Armstrong, Jim Burn, Ashley Ingram, Paula Long, Joe Phipps, Lewis Hare, Kirsty Walker, Charlotte Copley, James Keat, Craig Chedzoy, Leah Mason, David Willis, James King, Victoria Jones, Rich Godfrey, Martyn Morris, Dale Jefferies, Nicole Morrison, Kirsty Carpenter, Steve Palmer, Tara Marchant, Mark Reilly, Sabrina Bolwell, Matthew Adams, Helen Harris, Tracy Hoad, Stuart Stimson, Collette Young, Oscar Miller, Joe Meering, Ashley Watson, William Price, Katharine Laurenson, Lee Clarke, John Carrielies, Sharon Bowyer, Adam Blinman, Anna McCann, Carrie-Anne Durrant, Charlotte Blow, Chris Taplin, Marcus Goodwin, Damon Godfrey, Maree Holmes, Rhys Allsopp, Peter Willows, Katrina Scott, Sonia Wright-Stainton, Stacey Lawrence, Jacquiline Mullarney, Lewis Garrood, Andy Cole, Tom Rutherford, Luke Slocombe, Alan Piper, Julia Ryder, Tom Driver, Mike Smith, Hannah Barber, Joe Cartwright, Loxley Patterson, Chris Birch, Kelly Chappell, James Constable, Naomi Miller, Tara Rodda, Tim Taylor, Jason Trudgill, Russell Hoskin, Harry Brown, Julie

For peer review only - http://bmjopen.bmj.com/site/about/guidelines.xhtml

Legg, Toni Howell, Fergus Beeley, Zoe Flowerdew, Lucy Davies, Gary Rooney, Cassandra Day, Tamara Hoare, Dylan Carey, Lewis Murphy.

References:

1.Orejarena LA, Vidaillet H, DeStefano F, et al. Paroxysmal supraventricular tachycardia in the general population. J Am Coll Cardiol. 1998;31(1):150-157

2.Wood KA, Drew BJ, Scheinman MM. Frequency of disabling symptoms in supraventricular tachycardia. Am J Cardiol. 1997;79(2):145-9.

3. Smith G, McD Taylor D, Morgans A, Cameron P. Measuring the effectiveness of a revised clinical practice guideline for the pre-hospital management of supraventricular tachycardia. Emerg Med Australas. 2015 Feb;27(1):22-8. doi:10.1111/1742-6723.123404.

4. JRCALC Guidelines: https://aace.org.uk/clinical-practice-guidelines/

5. Resuscitation Council (UK) Resuscitation Guidelines 2005. http://www.resus.org.uk/pages/guide.htm

6. Smith G, Morgans, Boyle M. Use of the valsalva manoeuvre in the prehospital setting: a review of the literature. Emerg Med J. 2009;26:8-10

7. Honarbakhsh S, Baker V, Kirkby C, et al Safety and efficacy of paramedic treatment of regular supraventricular tachycardia: a randomised controlled trial Heart 2017;103:1413-1418.

8. Appelboam A, Green J, Ewings P, et al. Evaluation of Pre-hospital use of a Valsalva Assist Device in the Emergency treatment of supraventricular tachycardia [EVADE]: A randomised controlled feasibility trial. Pilot Feasibility Stud 6, 74 (2020). https://doi.org/10.1186/s40814-020-00616-y

9. Smith G, Boyle M. The 10ml syringe is useful in generating the recommended standard of 40mmHg intrathoracic pressure for the Valsalva manoeuvre. Emerg Med Aust 2009;21:449-454

For peer review only - http://bmjopen.bmj.com/site/about/guidelines.xhtml

10. European Cardiology Society SVT Guidelines https://www.escardio.org/Guidelines/Clinical-Practice-Guidelines/Supraventricular-Tachycardia Last accessed 27/11/22

11. Appelboam A, Reuben A, Mann C, et al; REVERT Trial Collaborators. Postural modification to the standard Valsalva manoeuvre for emergency treatment of supraventricular tachycardias (REVERT): a randomised controlled trial. Lancet 2015; 31;386:1747–53.

12. Thornton HS, Elwan MH, Reynolds JA, et al Valsalva using a syringe: pressure and variation Emerg Med J 2016;33:748-749.

13. FitzGerald I, Ewings P, Lang I, Appelboam A. Testing of a novel Valsalva Assist Device with supine and modified positions in healthy volunteers. Emerg Med J. 2019 Jan;36(1):27-31. doi: 10.1136/emermed-2018- 208004. Epub 2018 Dec 1

14. O'Cathain A, Knowles E, Bishop-Edwards L, et al. Understanding variation in ambulance service non-conveyance rates: a mixed methods study. Southampton (UK): NIHR Journals Library; 2018 May. (Health Services and Delivery Research, No. 6.19.) Available from: https://www.ncbi.nlm.nih.gov/books/NBK506846/doi: 10.3310/hsdr06190

15. Chan A-W, Tetzlaff JM, Gøtzsche PC, Altman DG, Mann H, Berlin J, Dickersin K, Hróbjartsson A, Schulz KF, Parulekar WR, Krleža-Jerić K, Laupacis A, Moher D. SPIRIT 2013 Explanation and Elaboration: Guidance for protocols of clinical trials. BMJ. 2013;346:e7586

16. NIHR. NIHR CRN Recruitment Policy Document Version 1.7 2018 [Available from: https://www.nihr.ac.uk/documents/nihr-crn-recruitment-policy-document/11347#21._Assumed_consent accessed 11/10/2021

17. Hussey MA, Hughes JP: Design and analysis of stepped-wedge cluster randomized trials. Contemp Clin Trials 2007, 28:182– 21. 91 Involve Guidelines https://www.invo.org.uk/ Last accessed 15/05/20

For peer review only - http://bmjopen.bmj.com/site/about/guidelines.xhtml

18. https://www.swast.nhs.uk/welcome/research-in-south-western-ambulance-service/evade/ Last accessed 03/01/23

19. Involve Guidelines https://www.invo.org.uk/ Last accessed 15/05/20

20. Mathews M, Parast L, Tolpadi A, et al. “Methods for Improving Response Rates in an Emergency Department Setting – A Randomized Feasibility Study.” Survey Practice 12 (1). https://doi.org/10.29115/SP-2019-0007.

Figures:

Figure 1. The ‘Valsa-Valve’ Valsalva Assist Device packaging and device details
Figure 2. Scheme for the Randomised Stepped Wedge Introduction of the VAD to the Ambulance Service

For peer review only

For peer review only - http://bmjopen.bmj.com/site/about/guidelines.xhtml

Figure 1. The 'Valsa-Valve' Valsalva Assist Device packaging and device details

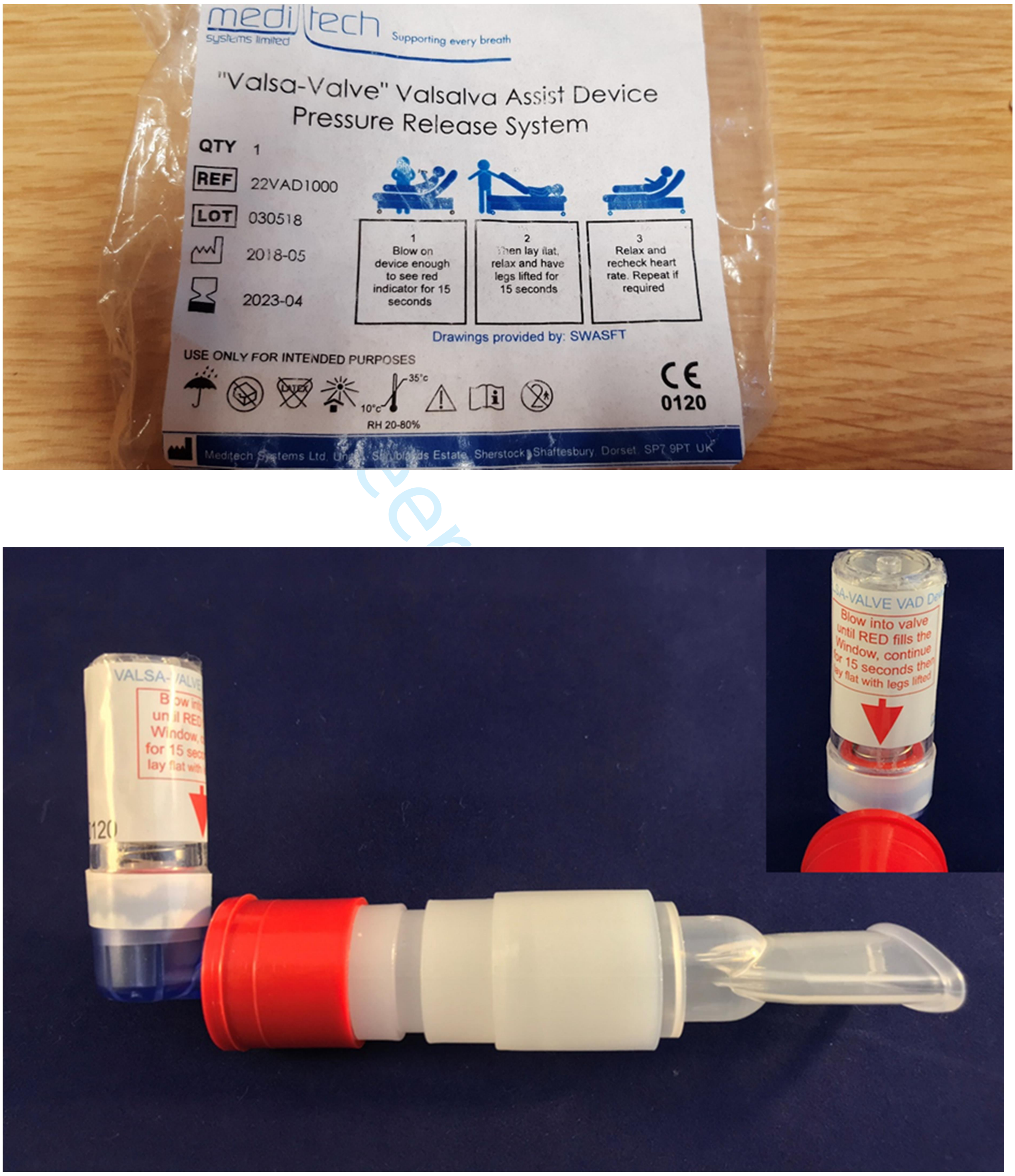

Figure 2. Scheme for the Randomised Stepped Wedge Introduction of the VAD to the Ambulance Service

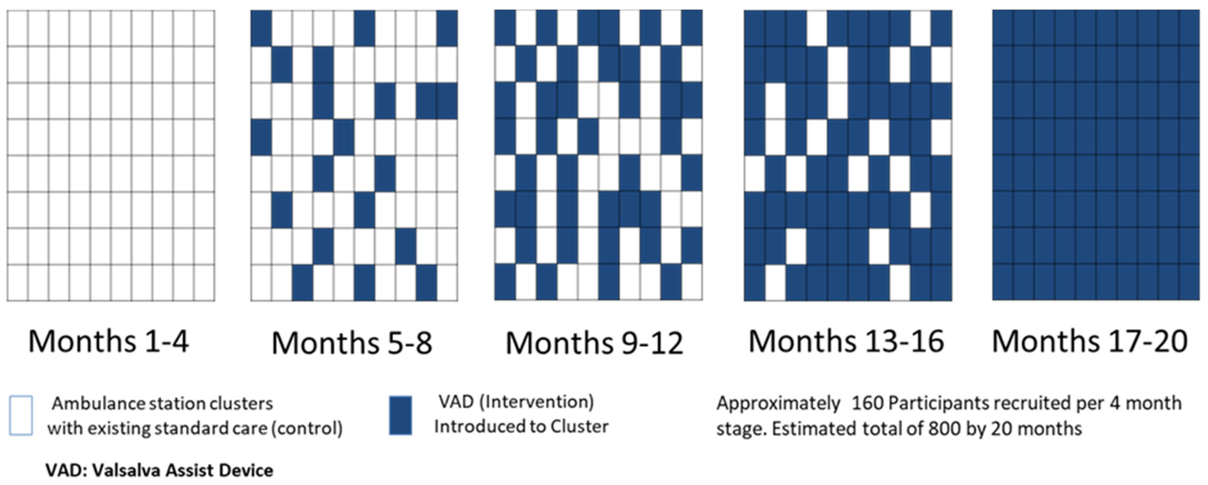

For peer review only

Reporting checklist for protocol of a clinical trial.

Based on the SPIRIT guidelines.

Instructions to authors

Complete this checklist by entering the page numbers from your manuscript where readers will find each of the items listed below.

Your article may not currently address all the items on the checklist. Please modify your text to include the missing information. If you are certain that an item does not apply, please write "n/a" and provide a short explanation.

Upload your completed checklist as an extra file when you submit to a journal.

In your methods section, say that you used the SPIRITreporting guidelines, and cite them as:

Chan A-W, Tetzlaff JM, Gøtzsche PC, Altman DG, Mann H, Berlin J, Dickersin K, Hróbjartsson A, Schulz KF, Parulekar WR, Krleža-Jerić K, Laupacis A, Moher D. SPIRIT 2013 Explanation and Elaboration: Guidance for protocols of clinical trials. BMJ. 2013;346:e7586

		Reporting Item	Page Number
**Administrative information**			
Title	#1	Descriptive title identifying the study design, population, interventions, and, if applicable, trial acronym	1

For peer review only

Trial registration	#2a	Trial identifier and registry name. If not yet registered, name of intended registry	2
Trial registration: data set	#2b	All items from the World Health Organization Trial Registration Data Set	2
Protocol version	#3	Date and version identifier	21
Funding	#4	Sources and types of financial, material, and other support	21
Roles and responsibilities: contributorship	#5a	Names, affiliations, and roles of protocol contributors	1 & 20
Roles and responsibilities: sponsor contact information	#5b	Name and contact information for the trial sponsor	1
Roles and responsibilities: sponsor and funder	#5c	Role of study sponsor and funders, if any, in study design; collection, management, analysis, and interpretation of data; writing of the report; and the decision to submit the report for publication, including whether they will have ultimate authority over any of these activities	21
Roles and responsibilities: committees	#5d	Composition, roles, and responsibilities of the coordinating centre, steering committee, endpoint adjudication committee, data management team, and	20

For peer review only

		other individuals or groups overseeing the trial, if applicable (see Item 21a for data monitoring committee)	
**Introduction**			
Background and rationale	#6a	Description of research question and justification for undertaking the trial, including summary of relevant studies (published and unpublished) examining benefits and harms for each intervention	4-5
Background and rationale: choice of comparators	#6b	Explanation for choice of comparators	4-5
Objectives	#7	Specific objectives or hypotheses	5
Trial design	#8	Description of trial design including type of trial (eg, parallel group, crossover, factorial, single group), allocation ratio, and framework (eg, superiority, equivalence, non-inferiority, exploratory)	5-16
**Methods: Participants, interventions, and outcomes**			
Study setting	#9	Description of study settings (eg, community clinic, academic hospital) and list of countries where data	5

For peer review only

		will be collected. Reference to where list of study sites can be obtained	
Eligibility criteria	#10	Inclusion and exclusion criteria for participants. If applicable, eligibility criteria for study centres and individuals who will perform the interventions (eg, surgeons, psychotherapists)	6
Interventions: description	#11a	Interventions for each group with sufficient detail to allow replication, including how and when they will be administered	5
Interventions: modifications	#11b	Criteria for discontinuing or modifying allocated interventions for a given trial participant (eg, drug dose change in response to harms, participant request, or improving / worsening disease)	7
Interventions: adherance	#11c	Strategies to improve adherence to intervention protocols, and any procedures for monitoring adherence (eg, drug tablet return; laboratory tests)	14
Interventions: concomitant care	#11d	Relevant concomitant care and interventions that are permitted or prohibited during the trial	7
Outcomes	#12	Primary, secondary, and other outcomes, including the specific measurement variable (eg, systolic blood pressure), analysis metric (eg, change from baseline, final value, time to event), method of aggregation (eg, median, proportion), and time point for each outcome.	13

For peer review only

		Explanation of the clinical relevance of chosen efficacy and harm outcomes is strongly recommended	
Participant timeline	#13	Time schedule of enrolment, interventions (including any run-ins and washouts), assessments, and visits for participants. A schematic diagram is highly recommended (see Figure)	8 & 12
Sample size	#14	Estimated number of participants needed to achieve study objectives and how it was determined, including clinical and statistical assumptions supporting any sample size calculations	13-14
Recruitment	#15	Strategies for achieving adequate participant enrolment to reach target sample size	14
**Methods: Assignment of interventions (for controlled trials)**			
Allocation: sequence generation	#16a	Method of generating the allocation sequence (eg, computer-generated random numbers), and list of any factors for stratification. To reduce predictability of a random sequence, details of any planned restriction (eg, blocking) should be provided in a separate document that is unavailable to those who enrol participants or assign interventions	9-11

For peer review only

Allocation concealment mechanism	#16b	Mechanism of implementing the allocation sequence (eg, central telephone; sequentially numbered, opaque, sealed envelopes), describing any steps to conceal the sequence until interventions are assigned	9-11
Allocation: implementation	#16c	Who will generate the allocation sequence, who will enrol participants, and who will assign participants to interventions	10
Blinding (masking)	#17a	Who will be blinded after assignment to interventions (eg, trial participants, care providers, outcome assessors, data analysts), and how	10
Blinding (masking): emergency unblinding	#17b	If blinded, circumstances under which unblinding is permissible, and procedure for revealing a participant's allocated intervention during the trial	10
**Methods: Data collection, management, and analysis**			
Data collection plan	#18a	Plans for assessment and collection of outcome, baseline, and other trial data, including any related processes to promote data quality (eg, duplicate measurements, training of assessors) and a description of study instruments (eg, questionnaires, laboratory tests) along with their reliability and validity,	15-16

For peer review only

		if known. Reference to where data collection forms can be found, if not in the protocol	
Data collection plan: retention	#18b	Plans to promote participant retention and complete follow-up, including list of any outcome data to be collected for participants who discontinue or deviate from intervention protocols	16
Data management	#19	Plans for data entry, coding, security, and storage, including any related processes to promote data quality (eg, double data entry; range checks for data values). Reference to where details of data management procedures can be found, if not in the protocol	16
Statistics: outcomes	#20a	Statistical methods for analysing primary and secondary outcomes. Reference to where other details of the statistical analysis plan can be found, if not in the protocol	15-16
Statistics: additional analyses	#20b	Methods for any additional analyses (eg, subgroup and adjusted analyses)	15-16
Statistics: analysis population and missing data	#20c	Definition of analysis population relating to protocol non-adherence (eg, as randomised analysis), and any statistical methods to handle missing data (eg, multiple imputation)	16

Methods: Monitoring

For peer review only

Data monitoring: formal committee	#21a	Composition of data monitoring committee (DMC); summary of its role and reporting structure; statement of whether it is independent from the sponsor and competing interests; and reference to where further details about its charter can be found, if not in the protocol. Alternatively, an explanation of why a DMC is not needed	16
Data monitoring: interim analysis	#21b	Description of any interim analyses and stopping guidelines, including who will have access to these interim results and make the final decision to terminate the trial	12-13, 14-15
Harms	#22	Plans for collecting, assessing, reporting, and managing solicited and spontaneously reported adverse events and other unintended effects of trial interventions or trial conduct	12-13
Auditing	#23	Frequency and procedures for auditing trial conduct, if any, and whether the process will be independent from investigators and the sponsor	10
**Ethics and dissemination**			
Research ethics approval	#24	Plans for seeking research ethics committee / institutional review board (REC / IRB) approval	16

For peer review only

Protocol amendments	#25	Plans for communicating important protocol modifications (eg, changes to eligibility criteria, outcomes, analyses) to relevant parties (eg, investigators, REC / IRBs, trial participants, trial registries, journals, regulators)	16
Consent or assent	#26a	Who will obtain informed consent or assent from potential trial participants or authorised surrogates, and how (see Item 32)	7-9
Consent or assent: ancillary studies	#26b	Additional consent provisions for collection and use of participant data and biological specimens in ancillary studies, if applicable	8
Confidentiality	#27	How personal information about potential and enrolled participants will be collected, shared, and maintained in order to protect confidentiality before, during, and after the trial	16
Declaration of interests	#28	Financial and other competing interests for principal investigators for the overall trial and each study site	21
Data access	#29	Statement of who will have access to the final trial dataset, and disclosure of contractual agreements that limit such access for investigators	1 & 21
Ancillary and post trial care	#30	Provisions, if any, for ancillary and post-trial care, and for compensation to those who suffer harm from trial participation	12 and covered by

For peer review only

			NHS indemnity
Dissemination policy: trial results	#31a	Plans for investigators and sponsor to communicate trial results to participants, healthcare professionals, the public, and other relevant groups (eg, via publication, reporting in results databases, or other data sharing arrangements), including any publication restrictions	16
Dissemination policy: authorship	#31b	Authorship eligibility guidelines and any intended use of professional writers	16
Dissemination policy: reproducible research	#31c	Plans, if any, for granting public access to the full protocol, participant-level dataset, and statistical code	21
**Appendices**			
Informed consent materials	#32	Model consent form and other related documentation given to participants and authorised surrogates	N/A
Biological specimens	#33	Plans for collection, laboratory evaluation, and storage of biological specimens for genetic or molecular analysis in the current trial and for future use in ancillary studies, if applicable	N/A

None The SPIRIT Explanation and Elaboration paper is distributed under the terms of the Creative Commons Attribution License CC-BY-NC. This checklist can be completed online using https://www.goodreports.org/, a tool made by the EQUATOR Network in collaboration with Penelope.ai

For peer review only
