# Supplementary material for: Evaluation of the prehospital use of a Valsalva assist device in the emergency treatment of supraventricular tachycardia (EVADE SVT): study protocol for a stepped wedge cluster randomised controlled trial
Source: BMJ Open. 2023 Jun 8;13(6):e073315. doi: 10.1136/bmjopen-2023-073315 (PMC10255124; doi:10.1136/bmjopen-2023-073315)
Supplement: Reviewer comments [file bmjopen-2023-073315.reviewer_comments.pdf]

PEER REVIEW HISTORY

BMJ Open publishes all reviews undertaken for accepted manuscripts. Reviewers are asked to complete a checklist review form (**http://bmjopen.bmj.com/site/about/resources/checklist.pdf**) and are provided with free text boxes to elaborate on their assessment. These free text comments are reproduced below.

ARTICLE DETAILS

**TITLE (PROVISIONAL)**	Evaluation of the pre-hospital use of a Valsalva assist device in the emergency treatment of supraventricular tachycardia (EVADE SVT): study protocol for a stepped wedge cluster randomised controlled trial
**AUTHORS**	Appelboam, Andrew; Osborne, Ria; Ukoumunne, Obioha; Black, Sarah; Boot, Suzanne; Richards, Nicolas; Scotney, Natalie; Rhodes, Shelley; Cranston, Tim; Hawker, Ruth; Gillett, Annette; Jones, Ben; Hawton, Annie; Dayer, Mark; Creanor, Siobhan; EVADE SVT study collaborators, EVADE SVT study collaborators

This article was not externally reviewed at BMJ Open. Protocols that have been independently assessed prior to submission to BMJ Open are usually fast-tracked to publication on the grounds that further substantial changes will not be possible. This independent assessment will usually be external, independent review for both a substantial grant award from a non-commercial or government funder and ethics approval.
